# Systematic Review and Meta-Analysis of Sex Differences in Social Contact Patterns and Implications for Tuberculosis Transmission and Control

**DOI:** 10.3201/eid2605.190574

**Published:** 2020-05

**Authors:** Katherine C. Horton, Anne L. Hoey, Guillaume Béraud, Elizabeth L. Corbett, Richard G. White

**Affiliations:** London School of Hygiene and Tropical Medicine, London, UK (K.C. Horton, E.L. Corbett, R.G. White);; St. George Hospital, Sydney, New South Wales, Australia (A.L. Hoey);; University of New South Wales, Sydney (A.L. Hoey);; Centre Hospitalier Universitaire de Poitiers, Poitiers, France (G. Béraud);; Université de Lille, Lille, France (G. Béraud);; Universiteit de Hasselt, Hasselt, Belgium (G. Béraud);; Malawi–Liverpool–Wellcome Trust Clinical Research Programme, Blantyre, Malawi (E.L. Corbett)

**Keywords:** sex differences, tuberculosis, tuberculosis and other mycobacteria, Mycobacterium tuberculosis, bacteria, social contact patterns, systematic review, mixing, meta-analysis, sex assortativity, transmission, control

## Abstract

Social contact patterns might contribute to excess burden of tuberculosis in men. We conducted a study of social contact surveys to evaluate contact patterns relevant to tuberculosis transmission. Available data describe 21 surveys in 17 countries and show profound differences in sex-based and age-based patterns of contact. Adults reported more adult contacts than children. Children preferentially mixed with women in all surveys (median sex assortativity 58%, interquartile range [IQR] 57%–59% for boys, 61% [IQR 60%–63%] for girls). Men and women reported sex-assortative mixing in 80% and 95% of surveys (median sex assortativity 56% [IQR 54%–58%] for men, 59% [IQR 57%–63%] for women). Sex-specific patterns of contact with adults were similar at home and outside the home for children; adults reported greater sex assortativity outside the home in most surveys. Sex assortativity in adult contacts likely contributes to sex disparities in adult tuberculosis burden by amplifying incidence among men.

Tuberculosis (TB) is the leading infectious cause of death worldwide; there were an estimated 1.3 million deaths during 2017 (*1*). Approximately 25% of the world’s population is infected with *Mycobacterium tuberculosis* (*2*), the bacterium that causes TB (*3*). Of 1.7 billion persons infected with *M. tuberculosis*, TB developed in 10 million persons during 2017 (*1*,*4*). Despite major investment in disease control efforts since the 1990s, progress has been slow; incidence is currently decreasing by only 1.5%/year (*3*).

TB predominantly affects men, who have 60% of reported cases and 65% of reported deaths globally (*1*). Men are less likely than women to access timely TB diagnosis and treatment (*5*,*6*) and remain infectious in the community for a much longer period (*5*,*7*). The impact is apparent from recent prevalence surveys of undiagnosed TB, which offer the most accurate measure of disease burden (*1*) and confirm pronounced sex disparity; men account for 70% of infectious cases in the community (*5*).

Critically, *M. tuberculosis* is spread person-to-person by airborne transmission. Undiagnosed infectious TB is the key driver of ongoing transmission, and most TB episodes reflect recent transmission from adult contacts (*3*). The excess burden of TB in men might be a result of broader socialization patterns that emerge during adolescence (*8*,*9*). The risk for TB in men might be amplified if sex-assortative (like-with-like by sex, male or female) mixing is prevalent, such that men have greater contact with other men than with women (*5*). Sex-specific social contact patterns might also be useful in understanding TB in women and children, as shown by analytical results suggesting most new *M. tuberculosis* infections among men, women, and children in South Africa and Zambia can be attributed to contact with men (*10*).

Data from social contact surveys provide insight into how individual behaviors drive disease dynamics at the population level (*11*), providing better predictions of patterns of infection for respiratory pathogens (*12*,*13*) than can be made from assumptions of homogenous or proportionate mixing (*14*). Several analyses have examined sex differences in social contact patterns, although most analyses report sex differences in the number of reported contacts. Only a few analyses have assessed the sex assortativity of contacts in sufficient detail to provide major insights into the transmission potential for diseases with major sex disparities, such as TB (*10*,*15*,*16*).

We conducted a systematic review and meta-analysis to examine sex differences in the number, sex assortativity, and location of social contacts reported by children and adults. Our main aims were to evaluate sex-based social contact patterns in children and adults, sex-assortative mixing among adults, and the frequency of contact between men and boys, men and girls, and men and women.

## Methods

### Search Strategy

We conducted this systematic review according to Preferred Reporting Items for Systematic Reviews and Meta-Analyses (PRISMA) (Appendix 1 Checklist 1) and Meta-Analyses of Observational Studies in Epidemiology (MOOSE) guidelines (Appendix 1 Checklist 2) in accordance with a published protocol (*17*). We identified publications describing social contact surveys conducted during January 1, 1997–August 5, 2018, through searches of PubMed, Embase, Global Health, and the Cochrane Database of Systematic Reviews (Appendix 1 Table 1). We searched reference lists from included publications by hand and contacted researchers with expertise in these surveys, particularly authors of a recent systematic review (*18*), to assist with identification of relevant publications.

Two authors (K.C.H. and A.L.H.) independently reviewed titles and then abstracts, in parallel, for relevance and included publications identified by either author for full-text review. These authors also reviewed full texts to determine which publications met inclusion criteria and then reviewed texts and supplemental materials to determine whether data on sex were recorded for participants and contacts. These authors contacted publication authors if it was unclear whether these data had been collected.

K.C.H. extracted data on methods from included surveys by using a piloted electronic form and gathered datasets from supplemental materials or a social contact data repository (https://www.socialcontactdata.org) if results were not reported in a format necessary for meta-analyses. When datasets were not publicly available, K.C.H contacted authors and asked them to share relevant results or data.

### Inclusion and Exclusion Criteria

The review included cross-sectional surveys conducted to assess social contact patterns relevant to airborne disease transmission that recorded participant sex and contact sex. We included only surveys that recorded all contacts over the survey period; we excluded surveys that examined only a subset of participants’ contacts (e.g., only those within a workplace or with other participants). We also excluded surveys that included only participants or contacts of a single sex and, because of limited sources for translation, publications in languages other than English. When we identified >1 report for a single survey, we included the earliest source or most complete dataset and excluded other records.

### Survey Quality

We assessed each survey by using the Appraisal Tool for Cross-sectional Studies (AXIS tool). This tool evaluates survey design, reporting quality, and risk for bias (*19*).

### Definitions

We considered participation equitable by sex if each sex made up 45%–55% of the survey population. We adjusted numbers of participants for analyses of physical and location-based contacts to exclude participants who did not report this information.

We stratified participants and contacts by age as children (boys and girls) and adults (men and women). For most surveys, adults were defined as persons >15 years of age (*1*); in instances where aggregate age categories did not enable disaggregation at this cutoff point, we used the nearest possible value. We defined close contacts, including physical and nonphysical contacts, according to survey-specific definitions, typically by a conversation longer than a greeting or >3 words.

We defined sex-assortative mixing as like-with-like contacts according to sex (male or female), either within age groups (e.g., men-with-men) or between age groups (e.g., men-with-boys). We defined preferential mixing as more mixing with 1 sex/age group than another.

### Data Analysis

For each survey, we calculated the average number of contacts over a 24-hour period for each sex/age category of participants with each sex/age category of contacts. For surveys in which data were collected over a 48-hour period, we divided the number of contacts by 2. For surveys in which data were collected over a 72-hour period, we divided the number of contacts by 3. We compared the average number of contacts across sex and age groups by using the Mann–Whitney–Wilcoxon test.

We calculated the percentage of sex-assortative mixing with 95% Clopper-Pearson CIs as contacts with the same sex divided by total contacts. We assessed sex-assortative mixing in children’s contacts with children and adults and in adults’ contacts with children and adults. We also compared the proportion of sex-assortative mixing by contact location: contacts within the home and contacts outside the home and, among contacts outside the home, contacts at work (for adults), school (for children), and elsewhere. We assessed heterogeneity by using the I^2^ statistic (*20*) and summarized findings across surveys by using the median and interquartile range (IQR).

We estimated the percentage of boys’, girls’, men’s and women’s adult contacts with men for subgroups based on survey setting characteristics (region, setting, and TB burden) and survey methods (sampling methods, reporting duration, age cutoff values for adults, and participation by sex). We excluded contact events for which the participant’s sex or age or the contact’s sex or age was missing. We made no adjustments for nonparticipation or nonsampling and used no weighting. We performed all analyses by using R version 3.2.2 (*21*).

## Results

**Figure 1:**
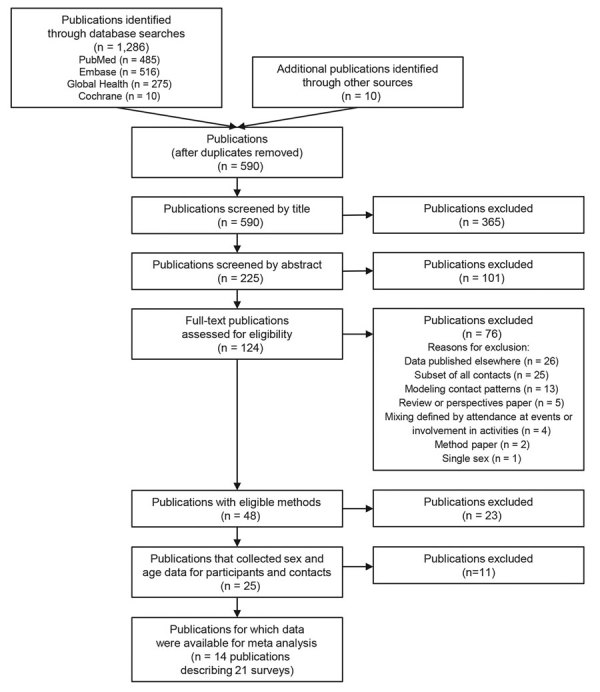
Preferred reporting items for systematic reviews and meta-analyses flowchart used for analysis of sex differences in social contact patterns and tuberculosis transmission and control.

Of 124 full-text publications reviewed for eligibility, we excluded 76 (Appendix 1 Table 2), and identified 48 that had eligible methods (Figure 1). Twenty-three publications described surveys that did not, to our knowledge, record sex and age for participants and contacts (Appendix 1 Table 3); 25 publications described surveys that were known to have recorded sex and age for participants and contacts (Appendix 1 Table 4). Data were available for meta-analysis from 14 publications describing 21 surveys (*10*,*13*–*16*,*22*–*30*) (Table; Appendix 2).

**Table:** Characteristics of 21 surveys of sex differences in social contact patterns and tuberculosis transmission and control

Survey region and country	Year(s)	Setting	Reporting period, h	Contact definition	Age of adults, y		No. participants					Reference
					Participants	Contacts	Men	Women	Boys	Girls	Total	
Africa												
South Africa	2010	Township	24	“physical touch” or “a 2-way conversation with 3 or more words in the physical presence of another person without physical touch”	>15	>15	186	191	92	102	571	(*25*)
South Africa	2011	8 communities in Western Cape	24	“face-to-face conversation that was longer than a greeting and within an arm’s reach”	>18	>13	634	636	0	0	1,270	(*10*)
Zambia	2011	16 communities	24	“face-to-face conversation that was longer than a greeting and within an arm’s reach”	>18	>13	1,018	1,124	0	0	2,142	(*10*)
Zimbabwe	2013	Manicaland	48	“interaction between two individuals, either physical (when involving skin-to-skin contact), or non-physical (when involving a two-way conversation with three or more words in the physical presence of another person, but no skin-to-skin contact)”	>13	>13	345	226	290	241	1,102	(*26*)
Americas												
Peru	2011	San Marcos	24	“a conversation with another person that is physically present and no farther than 3 meters, or a physical contact involving skin-to-skin touching, e.g., a kiss or handshake (either with or without conversation)”	>15	>15	132	156	135	135	558	(*23*)
Europe												
Belgium	2005–2006	National	24	“either skin-to-skin contact such as a kiss or handshake (a physical contact), or a two-way conversation with three or more words in the physical presence of another person but no skin-to-skin contact (a nonphysical contact)”	>15	>15	238	290	111	106	745	(*14*)
Belgium	2010–2011	Flanders	24	“a two-way conversation with a dialog of at least 3 words and skin-to-skin touching either with or without conversation.”	>15	>15	620	783	192	152	1,747	(*27*)
Finland	2005–2006	National	24	“either skin-to-skin contact such as a kiss or handshake (a physical contact), or a two-way conversation with three or more words in the physical presence of another person but no skin-to-skin contact (a nonphysical contact)”	>15	>15	327	362	155	131	975	(*14*)
France	2012	National	48	“talking to someone within a distance of less than 2 meters, or skin-to skin touching”	>15	>15	450	668	316	310	1,744	(*15*)
Germany	2005–2006	National	24	“either skin-to-skin contact such as a kiss or handshake (a physical contact), or a two-way conversation with three or more words in the physical presence of another person but no skin-to-skin contact (a nonphysical contact)”	>15	>15	426	553	137	144	1,260	(*14*)
Italy	2005–2006	National	24	“either skin-to-skin contact such as a kiss or handshake (a physical contact), or a two-way conversation with three or more words in the physical presence of another person but no skin-to-skin contact (a nonphysical contact)”	>15	>15	260	325	143	109	837	(*14*)
Luxembourg	2005–2006	National	24	“either skin-to-skin contact such as a kiss or handshake (a physical contact), or a two-way conversation with three or more words in the physical presence of another person but no skin-to-skin contact (a nonphysical contact)”	>15	>15	330	414	158	147	1,049	(*14*)
The Netherlands	2005–2006	National	24	“either skin-to-skin contact such as a kiss or handshake (a physical contact), or a two-way conversation with three or more words in the physical presence of another person but no skin-to-skin contact (a nonphysical contact)”	>15	>15	245	285	115	128	773	(*16**)*
Poland	2005–2006	National	24	“either skin-to-skin contact such as a kiss or handshake (a physical contact), or a two-way conversation with three or more words in the physical presence of another person but no skin-to-skin contact (a nonphysical contact)”	>15	>15	341	370	156	143	1,010	(*14*)
United Kingdom	2005–2006	National	24	“either skin-to-skin contact such as a kiss or handshake (a physical contact), or a two-way conversation with three or more words in the physical presence of another person but no skin-to-skin contact (a nonphysical contact)”	>15	>15	339	368	144	154	1,005	(*14*)
United Kingdom	2012	National	24	“an interaction in close proximity with three or more words directed to the infant or a physical skin-to-skin contact between infant and another person”	Not available	>15	0	0	62	53	115	(*28*)
Western Pacific												
Australia	2008	Greater Melbourne	72	“two-way or small group conversational exchange of at least 3 words, or any skin-to-skin contact”	>20	>15	11	54	0	0	65	(*29*)
Australia	2013	Greater Melbourne	24	“two-way face-to-face conversation of more than three words or any physical contact”	>18	>15	490	750	0	0	1,240	(*30*)
China	2010	Taiwan	24	“physical contacts and those nonphysical contacts with verbal communication made within 2 meters”	>15	>15	807	801	183	152	1,943	(*22*)
China	2015–2016	Hong Kong	24	“skin-to-skin touch such as a handshake (a physical contact) or a face-to-face conversation with three or more words in the physical presence of both the participant and the contact within two meters”	>15	>15	435	461	116	123	1,135	(*13*)
Vietnam	2007	Semirural community in Red River Delta	24	“either skin-to-skin contact (a physical contact), or a two-way conversation with three or more words in the physical presence of another person but no skin-to-skin contact”	>16	>16	264	346	125	125	860	(*24*)

Included surveys had >22,146 participants and 270,308 sex-specific/age-specific contacts. Surveys were conducted in 17 countries: 4 surveys with 5,085 participants in Africa, 1 survey with 558 participants in the Americas, 11 surveys with 11,260 participants in Europe, and 5 surveys with 5,243 participants in the Western Pacific region. Thirteen surveys were conducted in high-income countries, 5 in upper-middle-income countries, 2 in lower-middle-income countries, and 1 in a low-income country. Ten surveys were conducted at a national scale; 11 were subnational. All surveys were during 2005–2016. Seventeen surveys included child participants; 20 adult participants, and 16 both children and adults.

### Participation by Sex

Participation by children was considered equitable by sex in 15 (88%) of 17 surveys. In 2 (12%) surveys, participation by boys substantially exceeded that by girls; boys made up 56% and 57% of the population of each survey. Participation by adults was considered equitable by sex in 11 (55%) of 20 surveys. In 8 (40%) of 20 surveys, participation by women substantially exceeded that by men; women made up 56%–83% of the population of each survey. In 1 (5%) survey, participation by men substantially exceeded that by women; men made up 60% of the survey population.

### Social Contacts by Boys and Girls

The median number of contacts reported over a 24-hour period was 12.9 (IQR 9.3–15.9) for boys and 13.5 (IQR 9.5–15.9) for girls (Appendix 1 Table 5); the difference in numbers of contacts was not significant (p = 0.92). Approximately half of contacts reported by boys (median 53%, IQR 43%–55%) and girls (median 51%, IQR 45%–56%) were with other children.

**Figure 2:**
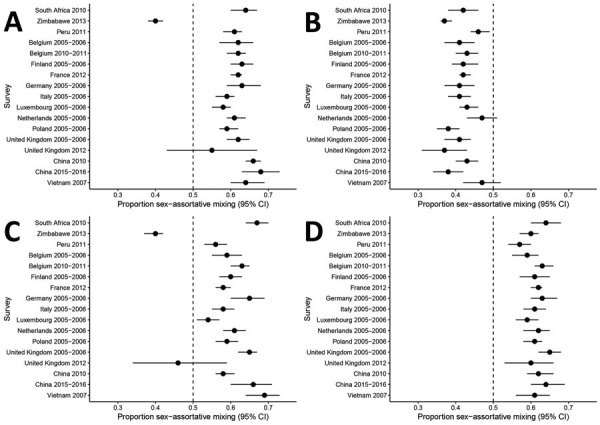
Analysis of sex differences in social contact patterns and tuberculosis transmission and control showing proportion of contacts with the same sex as reported for A) boys with boys, B) boys with men, C) girls with girls, and D) girls with women. Forest plots of sex-assortative mixing in contacts show contacts (black dots) and 95% CIs (error bars) reported by boys (A, B) and girls (C, D) with children (A, C) and with adults (B, D).

Among contacts of children with other children, we found strong evidence of sex-assortative mixing reported by boys in 15 (88%) of 17 surveys and by girls in 15 (88%) of 17 surveys (Figure 2, panels A, C; Appendix 1 Table 6). The median percentage of sex-assortative mixing in contacts with children was 62% (IQR 59%–63%) for boys and 59% (IQR 59%–65%) for girls. Summary measures are not reported because of substantial heterogeneity between surveys (I^2^ = 96.3% for boys, I^2^ = 95.6% for girls).

Among contacts of children with adults, there was no evidence of sex-assortative mixing reported by boys and strong evidence reported by girls in 17 (100%) of 17 surveys (Figure 2, panel B, D, Appendix 1 Table 6). The median percentage of sex-assortative mixing was 42% (IQR 41%–43%) for boys and 61% (IQR 60%–63%) for girls. Boys reported preferential mixing with women in 15 (88%) of 17 surveys. Summary measures are not reported because of substantial heterogeneity between surveys (*I*^2^ = 73.8% for boys, *I*^2^ = 44.3% for girls).

**Figure 3:**
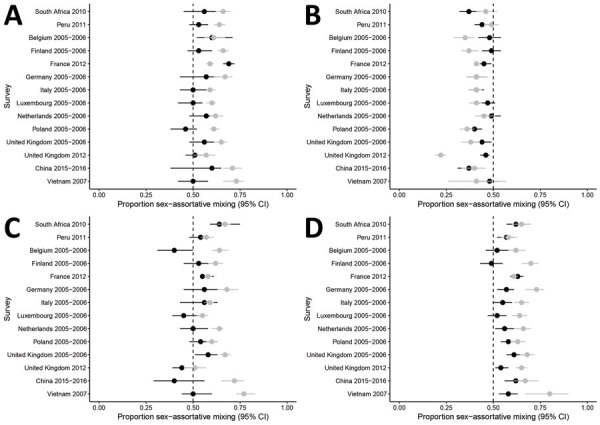
Analysis of sex differences in social contact patterns and tuberculosis transmission and control showing proportion of contacts with the same sex, disaggregated by location, as reported for A) boys with boys, B) boys with men, C) girls with girls, and D) girls with women. Forest plots of sex-assortative mixing show contacts at home (black dots) and outside the home (gray dots) with 95% CIs (error bars) reported by boys (A, B) and girls (C, D) with children (A, C) and with adults (B, D).

Most contacts reported by children took place outside the home (median 65% [IQR 62%–72% for boys], median 67% [IQR 56%–73%] for girls) (Appendix 1 Table 7). The sex assortativity of children’s contacts outside the home was similar to that at home. Among contacts with children, boys and girls reported more sex-assortative mixing in contacts outside the home than at home in 6 (43%) of 14 surveys for boys and 5 (36%) of 14 surveys for girls (Figure 3, panels A, C; Appendix 1 Table 8). Among contacts with adults, boys reported no more sex-assortative mixing in adult contacts outside the home than at home in 14 (100%) of 14 (100%) surveys, and girls reported more sex-assortative mixing outside the home than at home in 6 (42%) of 14 surveys (Figure 3, panels B, D; Appendix 1 Table 8). Summary measures are not reported because of substantial heterogeneity between surveys (*I*^2^ = 88.4% for boys, *I*^2^ = 83.0% for girls).

Among contacts of children outside the home, ≈50% of contacts of boys and girls contacts (median 56% [IQR 39%–62%] for boys, median 55% [IQR 38%–63%] for girls) occurred at school (Appendix 1 Table 9). We found few differences in the sex assortativity of contacts at school compared with those at other locations outside the home (Appendix 1 Table 10, Figure 1). Summary measures are not reported because of substantial heterogeneity between surveys (*I*^2^ = 84.7% for boys, *I*^2^ = 74.1% for girls).

### Social Contacts by Men and Women

The median number of contacts reported over a 24-hour period was 11.1 (IQR 8.1–15.3) for men and 11.6 (IQR 7.8–14.3) for women (Appendix 1 Table 11); the differences were not significant (p = 0.88), and the total number of contacts reported by adults did not differ from the total number of contacts reported by children (p = 0.26). Most contacts reported by men (median 91% [IQR 88%–93%] and women (median 87% [IQR 83%–90%]) were with other adults, which was significantly more than the number of adult contacts reported by children (p = 0.01).

**Figure 4:**
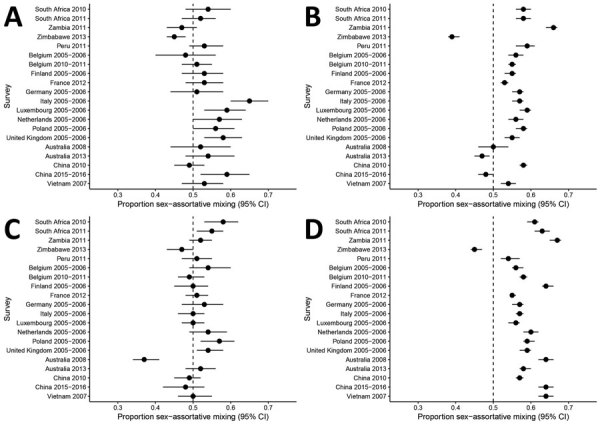
Analysis of sex differences in social contact patterns and tuberculosis transmission and control showing proportion of contacts with the same sex as reported for A) men with boys, B) men with men, C) women with girls, and D) women with women. Forest plots of sex-assortative mixing in contacts show contacts (black dots) and 95% CIs (error bars) reported by men (A, B) and women (C, D) with children (A, C) and with adults (B, D).

Among contacts of adults with children, there was strong evidence of sex-assortative mixing reported by men in 4 (20%) of 20 surveys and by women in 4 (20%) of 20 surveys (Figure 4, panels A, C; Appendix 1 Table 12). In 15 (75%) of 20 surveys, there was no major evidence of preferential mixing by sex reported by men or women in contacts with children. The median percentage of sex-assortative mixing was 53% (IQR 50%–57%) for men and 52% (IQR 50%–54%) for women. Summary measures are not reported because of substantial heterogeneity between surveys (*I*^2^ = 76.3% for boys, *I*^2^ = 81.6% for girls).

Among adult contacts with other adults, there was strong evidence of sex-assortative mixing reported by men in 16 (80%) of 20 surveys and by women in 19 (95%) of 20 surveys (Figure 4, panels B, D; Appendix 1 Table 12). The median percentage of sex-assortative mixing was 56% (IQR 54%–58%) for men and 59 (IQR 57%–63%) for women. Summary measures are not reported because of substantial heterogeneity between surveys (*I*^2^ = 98.1% for men, *I*^2^ = 97.0% for women).

**Figure 5:**
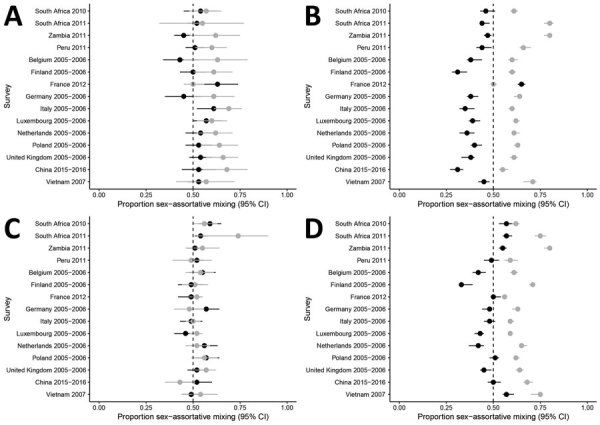
Analysis of sex differences in social contact patterns and tuberculosis transmission and control showing proportion of contacts with the same sex, disaggregated by location, as reported for A) men with boys, B) men with men, C) women with girls, and D) women with women. Forest plots of sex-assortative show mixing in contacts at home (black dots) and outside the home (gray dots) with 95% CIs (error bars) reported by men (A, B) and women (C, D) with children (A, C) and with adults (B, D) at home (black dots) and outside the home (gray dots).

Most contacts reported by adults took place outside the home (median 74%, IQR 62%–77% for men; median 70%, IQR 54%–76% for women) (Appendix 1 Table 13). Contacts of adults with children showed similar sex assortativity at home and outside the home (Figure 5, panels A, C; Appendix 1 Table 14). Among contacts of adults with adults, there was more sex-assortative mixing by men and women in contacts outside the home than in contacts within the home in 14 (93%) of 15 surveys (Figure 5, panel B, D; Appendix 1 Table 14). Summary measures are not reported because of substantial heterogeneity between surveys (*I*^2^ = 63.1% for men, *I*^2^ = 28.6% for women).

Among adult contacts outside the home, ≈33% of contacts of men and women (median 35% [IQR 28%–39%] for men, median 29% [IQR 26%–34%] for women) occurred at work (Appendix 1 Table 15). Because adults reported few contacts with children at work, CIs are wide for sex-assortative mixing estimates for men and women in most surveys (Appendix 1 Table 16, Figure 2, panels A, C). Men reported more sex-assortative mixing in contacts with other adults at work compared with contacts elsewhere outside the home in 12 (80%) of 15 surveys and elsewhere in 1 (7%) of 15 surveys (Appendix 1 Table 16, Figure 2, panels B, D). Women reported more sex-assortative mixing at work compared with contacts elsewhere outside the home in only 2 (13%) of 15 surveys and elsewhere in 1 (7%) of 15 surveys. Summary measures are not reported because of substantial heterogeneity between surveys (*I*^2^ = 32.3% for men, *I*^2^ = 87.0% for women).

### Subgroup Analyses

Subgroup analyses did not show clear differences in the frequency of contact with men by survey setting or method. There was little variation in survey characteristics measured by the AXIS tool (Appendix 1 Table 17). Substantial heterogeneity remained in summary measures for subgroups examined (Appendix 1 Table 18).

## Discussion

The main finding of this systematic review and meta-analysis of 21 social contact surveys in 17 countries is that sex differences in social contact patterns are profound, to an extent likely to be amplifying sex disparities in the adult burden of TB in many settings. Differences in sex-specific and age-specific social contact patterns between children and adults suggest a behavioral shift during adolescence, potentially driving the emergence of sex difference in TB epidemiology in adults. Sex-assortative mixing in adult contacts was reported by men in 80% of surveys and women in 95% of surveys. These findings have critical implications for men’s health and for broader TB prevention efforts because half of men’s contacts, one third of women’s contacts, and one fifth of children’s contacts were with adult men.

Social contact patterns clearly differ for children and adults. There was no major difference in the total number of contacts reported by children and adults. However, half of children’s contacts were with other children, who are less likely than adults to have TB or to transmit *M. tuberculosis* (*31*), and most adult contacts were with other adults. Children of both sexes frequently reported preferential mixing with women in adult contacts, and men and women both reported sex assortativity in contacts with other adults.

Among children, sex-specific patterns of contact with adults were similar at home and outside the home, and preferential mixing with women was reported across locations. Although many contacts were reported at school and substantial child contact time occurs at school (*25*), those contacts include few adult contacts and therefore limited opportunity for exposure to *M. tuberculosis*. These differences in contact patterns among children and adults support recent genetic epidemiology studies suggesting that only a small proportion of adult infections occur within the household (*32*,*33*) but that the odds of household transmission of *M. tuberculosis* are much higher among children (*34*). The higher number of adult contacts outside the home and greater sex assortativity of those contacts compared with children might partially explain the emergence of sex differences in TB epidemiology in adults.

In nearly all of the surveys examined, strong sex-assortative mixing in adult contacts was reported by men and women, as noted in previous studies that have examined sex assortativity (*10*,*15*,*16*). Results from our study indicate that in many settings, sex-assortative mixing might exacerbate the disproportionate burden of disease for men by amplifying risk for infection in a population already at greater risk for disease because of a nexus of biological, sociobehavioral, and health systems factors (*5*). Further research is needed to determine the relative contribution of sex-assortative mixing among these factors.

Among adults, reports of sex-assortative mixing were not symmetric; men reported less sex-assortative mixing than women in nearly half of surveys conducted among adults. In 3 surveys in which men did not report strong sex-assortative mixing, women did (*13*,*29*,*30*), raising questions of reporting bias. Previous studies that used wireless sensor devices have shown greater concordance between sensor and self-report methods for women than men (*35*), suggesting that inconsistencies might, in part, reflect less accurate reporting by men.

Only 1 survey, from rural and periurban Zimbabwe, reported no assortative mixing by adult respondents (*26*). This survey provided strong evidence of true negative sex assortativity among boys, girls, men, and women, suggesting underlying differences in social behavior that affect social interactions might pertain in some settings. This survey was similar in design to other surveys, but also reported a young age structure and substantial intergenerational mixing with extremes of age (*26*). Sex differences were less pronounced in the 2014 national TB survey in Zimbabwe than in other countries in Africa (*1*).

Our analysis of social contact patterns across sex and age groups has implications for *M. tuberculosis* transmission beyond understanding the excess burden of TB in men. Although sex-assortative mixing among adults to some extent protects women from exposure to *M. tuberculosis* transmission, one third of women’s contacts and one fifth of children’s contacts were with men. Therefore, the excess burden of TB among men has implications for *M. tuberculosis* transmission across the population, making strategies to provide early diagnosis of TB for men of potentially high public health value.

Our study had several limitations. Less than half of eligible publications had data on sex and age for participants and contacts, limiting the number of surveys included in our analyses. We recommend that future social contact surveys collect and report these data, ideally by using standardized tools to try to reduce high intersurvey heterogeneity that prevented us from reporting summary measures. In addition, our focus on close contacts will have excluded some contacts relevant to the spread of *M. tuberculosis* (*36*) but was dictated by data availability because no surveys reported casual contacts by sex. We also did not assess the intimacy or duration of contacts by sex.

Our analysis in only 2 age categories (children and adults) also reflects the nature of available data but might have led us to overlook more nuanced age differences in sex-based social contact patterns. Some surveys deliberately oversampled certain age groups, and we made no adjustments in our analyses for sampling bias and used no weighting, because of a lack of data on which to weight. Response bias might also have affected results, but few surveys reported the response rate, and none distinguished the response rate by sex.

Men are often overlooked in discussions of sex and TB, and strategies to assess and address men’s excess burden of disease and barriers to TB care are notably absent from the global research agenda. However, because men have most TB cases and remain untreated, and therefore infectious, longer than women, a better understanding of the factors that drive their disproportionate burden of disease is essential to appropriately direct resources to address these disparities. Our results show that social contact patterns likely contribute to the emergence of sex disparities in the adult burden of TB by amplifying men’s burden of disease. Contacts of men with women, boys, and girls show that the excess burden of TB among men also has serious implications for *M. tuberculosis* transmission across sex and age groups. Addressing the excess burden of TB in men is essential to improve men’s health and to meet the ambitious targets for reducing TB incidence and deaths (*37*,*38*).

Appendix 1 Additional information on systematic review and meta-analysis of sex differences in social contact patterns and implications for tuberculosis transmission and control.

Appendix 2 Additional information (definitions and dataset) on systematic review and meta-analysis of sex differences in social contact patterns and implications for tuberculosis transmission and control.
