# Supplementary material for: Systematic Review and Meta-Analysis of Sex Differences in Social Contact Patterns and Implications for Tuberculosis Transmission and Control
Source: Emerg Infect Dis. 2020 May;26(5):910–9. doi: 10.3201/eid2605.190574 (PMC7181919; doi:10.3201/eid2605.190574)
Supplement: Appendix 1 — Additional information on systematic review and meta-analysis of sex differences in social contact patterns and implications for tuberculosis transmission and control. [file 19-0574-Techapp-s1.pdf]

Article DOI: https://doi.org/10.3201/eid2605.190574

Sex Differences in Social Contact Patterns and Tuberculosis Transmission and Control

Appendix 1

Appendix 1 Checklist 1. PRISMA Checklist

Section/topic	#	Checklist item	Reported in section and paragraph or page no.
		**TITLE**	
Title	1	Identify the report as a systematic review, meta-analysis, or both.	Title
		**ABSTRACT**	
Structured summary	2	Provide a structured summary including, as applicable: background; objectives; data sources; study eligibility criteria, participants, and interventions; study appraisal and synthesis methods; results; limitations; conclusions and implications of key findings; systematic review registration number.	Abstract (as possible within journal word limits)
		**INTRODUCTION**	
Rationale	3	Describe the rationale for the review in the context of what is already known.	Introduction par. 1-4
Objectives	4	Provide an explicit statement of questions being addressed with reference to participants, interventions, comparisons, outcomes, and study design (PICOS).	Introduction par. 5
		**METHODS**	
Protocol and registration	5	Indicate if a review protocol exists, if and where it can be accessed (e.g., Web address), and, if available, provide registration information including registration number.	Methods par. 1
Eligibility criteria	6	Specify study characteristics (e.g., PICOS, length of follow-up) and report characteristics (e.g., years considered, language, publication status) used as criteria for eligibility, giving rationale.	Methods par. 1
Information sources	7	Describe all information sources (e.g., databases with dates of coverage, contact with study authors to identify additional studies) in the search and date last searched.	Methods par. 1
Search	8	Present full electronic search strategy for at least one database, including any limits used, such that it could be repeated.	Appendix 1 Table 1
Study selection	9	State the process for selecting studies (i.e., screening, eligibility, included in systematic review, and, if applicable, included in the meta-analysis).	Methods par. 2, 4
Data collection process	10	Describe method of data extraction from reports (e.g., piloted forms, independently, in duplicate) and any processes for obtaining and confirming data from investigators.	Methods par.3
Data items	11	List and define all variables for which data were sought (e.g., PICOS, funding sources) and any assumptions and simplifications made.	Methods par. 6-11
Risk for bias in individual studies	12	Describe methods used for assessing risk for bias of individual studies (including specification of whether this was done at the study or outcome level), and how this information is to be used in any data synthesis.	Methods par. 5
Summary measures	13	State the principal summary measures (e.g., risk ratio, difference in means).	Methods par. 9-11

Section/topic	#	Checklist item	Reported in section and paragraph or page no.
Synthesis of results	14	Describe the methods of handling data and combining results of studies, if done, including measures of consistency (e.g., I^2) for each meta-analysis.	Methods par. 9-11
Risk for bias across studies	15	Specify any assessment of risk for bias that may affect the cumulative evidence (e.g., publication bias, selective reporting within studies).	Not done
Additional analyses	16	Describe methods of additional analyses (e.g., sensitivity or subgroup analyses, meta-regression), if done, indicating which were pre-specified.	Methods par. 9-11
		**RESULTS**	
Study selection	17	Give numbers of studies screened, assessed for eligibility, and included in the review, with reasons for exclusions at each stage, ideally with a flow diagram.	Results par. 1
Study characteristics	18	For each study, present characteristics for which data were extracted (e.g., study size, PICOS, follow-up period) and provide the citations.	Appendix Table
Risk for bias within studies	19	Present data on risk for bias of each study and, if available, any outcome level assessment (see item 12).	Appendix 1 Table17
Results of individual studies	20	For all outcomes considered (benefits or harms), present, for each study: (a) simple summary data for each intervention group (b) effect estimates and confidence intervals, ideally with a forest plot.	Figures 2–5, Appendix 1
Synthesis of results	21	Present results of each meta-analysis done, including confidence intervals and measures of consistency.	Not done
Risk for bias across studies	22	Present results of any assessment of risk for bias across studies (see Item 15).	Not done
Additional analysis	23	Give results of additional analyses, if done (e.g., sensitivity or subgroup analyses, meta-regression [see Item 16]).	Appendix 1 Table18
		**DISCUSSION**	
Summary of evidence	24	Summarize the main findings including the strength of evidence for each main outcome; consider their relevance to key groups (e.g., healthcare providers, users, and policy makers).	Discussion par. 1-4,7,10
Limitations	25	Discuss limitations at study and outcome level (e.g., risk for bias), and at review-level (e.g., incomplete retrieval of identified research, reporting bias).	Discussion par. 8, 9
Conclusions	26	Provide a general interpretation of the results in the context of other evidence, and implications for future research.	Discussion par. 4, 5
		**FUNDING**	
Funding	27	Describe sources of funding for the systematic review and other support (e.g., supply of data); role of funders for the systematic review.	Funding statement

From: Moher D, Liberati A, Tetzlaff J, Altman DG, The PRISMA Group (2009). Preferred Reporting Items for Systematic Reviews and Meta-Analyses: The PRISMA Statement. PLoS Med 6(7): e1000097. doi:10.1371/journal.pmed1000097

Appendix 1 Checklist 2. MOOSE Checklist

Item No	Recommendation	Reported on Page No
Reporting of background should include		
1	Problem definition	Introduction par. 1-2
2	Hypothesis statement	Introduction par. 3
3	Description of study outcome(s)	Introduction par. 5
4	Type of exposure or intervention used	Not applicable
5	Type of study designs used	Methods par. 4
6	Study population	Methods par. 4
Reporting of search strategy should include		
7	Qualifications of searchers (e.g., librarians and investigators)	Methods par. 2
8	Search strategy, including time period included in the synthesis and keywords	Methods par. 1
9	Effort to include all available studies, including contact with authors	Methods par. 2, 3
10	Databases and registries searched	Methods par. 1
11	Search software used, name and version, including special features used (e.g., explosion)	Methods par. 1, Appendix 1 Table 1
12	Use of hand searching (e.g., reference lists of obtained articles)	Methods par. 1
13	List of citations located and those excluded, including justification	Appendix 1 Tables 2-4
14	Method of addressing articles published in languages other than English	Methods par. 4
15	Method of handling abstracts and unpublished studies	Not done
16	Description of any contact with authors	Methods par. 2, 3
Reporting of methods should include		
17	Description of relevance or appropriateness of studies assembled for assessing the hypothesis to be tested	Methods par. 4
18	Rationale for the selection and coding of data (e.g., sound clinical principles or convenience)	Methods par. 6-8
19	Documentation of how data were classified and coded (e.g., multiple raters, blinding and interrater reliability)	Methods par. 6-8
20	Assessment of confounding (e.g., comparability of cases and controls in studies where appropriate)	Not applicable
21	Assessment of study quality, including blinding of quality assessors, stratification or regression on possible predictors of study results	Methods par. 5
22	Assessment of heterogeneity	Methods par. 10

Item No	Recommendation	Reported on Page No
23	Description of statistical methods (e.g., complete description of fixed or random effects models, justification of whether the chosen models account for predictors of study results, dose-response models, or cumulative meta-analysis) in sufficient detail to be replicated	Methods par. 9-11
24	Provision of appropriate tables and graphics	Figures 2–5, Appendix 1
Reporting of results should include		
25	Graphic summarizing individual study estimates and overall estimate	Figures 2–5, Appendix 1
26	Table giving descriptive information for each study included	Appendix Table
27	Results of sensitivity testing (e.g., subgroup analysis)	Appendix 1 Table 18
28	Indication of statistical uncertainty of findings	Results par. 4-13

Appendix 1 Table 1. Search strategy

Set	PubMed	Embase/Global Health	Cochrane Library
1	(social contact*[Title/Abstract] OR contact pattern*[Title/Abstract] OR social mixing[Title/Abstract])	(social contact* or contact pattern* or social mixing).ab,ti.	(social contact* or contact pattern* or social mixing):ti,kw
2	(infectious disease*[Title/Abstract] OR respiratory[Title/Abstract] OR tuberculosis[Title/Abstract] OR influenza[Title/Abstract] OR transmission[Title/Abstract])	(infectious disease* or respiratory or tuberculosis or influenza or transmission).ab,ti.	(infectious disease* or respiratory or tuberculosis or influenza or transmission):ti,kw
3	“1997/01/01”[Date - Publication]: “3000”[Date - Publication]	1 and 2	(#1 AND #2)
4	English [la]	limit 3 to (English language and yr = ”1997 -Current”)	Limit 3 to time period 1997–present
5	1 AND 2 AND 3 AND 4		

Appendix 1 Table 2. Reasons for Exclusion of Publications After Full-text Review

Reference	Reason for Exclusion
Aiello AE, Simanek AM, Eisenberg MC, Walsh AR, Davis B, Volz E, et al. Design and methods of a social network isolation study for reducing respiratory infection transmission: The eX-FLU cluster randomized trial. Epidemics. 2016;15:38–55. doi: http://dx.doi.org/10.1016/j.epidem.2016.01.001. PubMed PMID: 608374678.	Participants report contacts only with other study participants
Alexander ME, Kobes R. Effects of vaccination and population structure on influenza epidemic spread in the presence of two circulating strains. BMC public health. 2011;11 Suppl 1:S8. PubMed PMID: 560051654.	Modeling study
Amaku M, Coutinho FA, Azevedo RS, Burattini MN, Lopez LF, Massad E. Vaccination against rubella: analysis of the temporal evolution of the age-dependent force of infection and the effects of different contact patterns. Physical review. 2003;E, Statistical, nonlinear, and soft matter physics. 67(5 Pt 1):051907. PubMed PMID: 137611835.	Modeling study
Andrews JR, Morrow C, Walensky RP, Wood R. Integrating social contact and environmental data in evaluating tuberculosis transmission in a South African township. Journal of Infectious Diseases. 2014;210(4):597–603. doi: http://dx.doi.org/10.1093/infdis/jiu138. PubMed PMID: 373710043.	Data published elsewhere (Johnstone Robertson 2011)
Apolloni A, Poletto C, Colizza V. Age-specific contacts and travel patterns in the spatial spread of 2009 H1N1 influenza pandemic. BMC Infectious Diseases. 2013;13 (1) (no pagination)(176). doi: http://dx.doi.org/10.1186/1471-2334-13-176. PubMed PMID: 52541688.	Data published elsewhere (Mossong 2008)
Bansal S, Read J, Pourbohloul B, Meyers LA. The dynamic nature of contact networks in infectious disease epidemiology. Journal of Biologic Dynamics. 2010;4(5):478–89. doi: http://dx.doi.org/10.1080/17513758.2010.503376. PubMed PMID: 362174279.	Review or perspectives piece
Barrat A, Cattuto C, Tozzi AE, Vanhems P, Voirin N. Measuring contact patterns with wearable sensors: Methods, data characteristics and applications to data-driven simulations of infectious diseases. Clinical Microbiology and Infection. 2014;20(1):10–6. doi: http://dx.doi.org/10.1111/1469-0691.12472. PubMed PMID: 370529746.	Participants report contacts only with other study participants
Benavides J, Demianyk BCP, Mukhi SN, Laskowski M, Friesen M, McLeod RD. Smartphone technologies for social network data generation and infectious disease modeling. Journal of Medical and Biologic Engineering. 2012;32(4):235–44. doi: http://dx.doi.org/10.5405/jmbe.974. PubMed PMID: 365841598.	Methodology paper
Blaser N, Zahnd C, Hermans S, Salazar-Vizcaya L, Estill J, Morrow C, et al. Tuberculosis in Cape Town: An age-structured transmission model. Epidemics. 2016;14:54–61. doi: http://dx.doi.org/10.1016/j.epidem.2015.10.001. PubMed PMID: 607220757.	Data published elsewhere (Johnstone Robertson 2011)
Campbell PT, McVernon J, Shrestha N, Nathan PM, Geard N. Who's holding the baby? A prospective diary study of the contact patterns of mothers with an infant. BMC Infectious Diseases. 2017;17 (1) (no pagination)(634). doi: http://dx.doi.org/10.1186/s12879-017-2735-8. PubMed PMID: 618339477.	Single sex participants (women)
Cauchemez S, Valleron AJ, Boelle PY, Flahault A, Ferguson NM. Estimating the impact of school closure on influenza transmission from Sentinel data. Nature. 2008;452(7188):750–4. doi: http://dx.doi.org/10.1038/nature06732. PubMed PMID: 351521077.	Modeling study
Chan TC, Fu YC, Hwang JS. Changing social contact patterns under tropical weather conditions relevant for the spread of infectious diseases. Epidemiology and Infection. 2015;143(2):440–51. doi: http://dx.doi.org/10.1017/S0950268814000843. PubMed PMID: 53155073.	Data published elsewhere (Fu 2012)
Chen SC, Chang CF, Jou LJ, Liao CM. Modeling vaccination programmes against measles in Taiwan. Epidemiology and Infection. 2007;135(5):775–86. doi: http://dx.doi.org/10.1017/S0950268806007369. PubMed PMID: 47161661.	Modeling study
Conlan AJK, Eames KTD, Gage JA, von Kirchbach JC, Ross JV, Saenz RA, et al. Measuring social networks in british primary schools through scientific engagement. Proceedings of the Royal Society B: Biologic Sciences. 2011;278(1711):1467–75. doi: http://dx.doi.org/10.1098/rspb.2010.1807. PubMed PMID: 361607401.	Participants report contacts only within school
Cornforth DM, Reluga TC, Shim E, Bauch CT, Galvani AP, Meyers LA. Erratic flu vaccination emerges from short-sighted behavior in contact networks. PLoS Computational Biology. 2011;7 (1) (no pagination)(e1001062). doi: http://dx.doi.org/10.1371/journal.pcbi.1001062. PubMed PMID: 361204748.	Modeling study
Danon L, Read JM, House TA, Vernon MC, Keeling MJ. Social encounter networks: characterizing Great Britain. Proceedings. 2013;Biologic sciences / The Royal Society. 280(1765):20131037. PubMed PMID: 563039898.	Data published elsewhere (Danon 2012)
De Cao E, Zagheni E, Manfredi P, Melegaro A. The relative importance of frequency of contacts and duration of exposure for the spread of directly transmitted infections. Biostatistics (Oxford, England). 2014;15(3):470–83. doi: http://dx.doi.org/10.1093/biostatistics/kxu008. PubMed PMID: 605882135.	Data published elsewhere (Mossong 2008)
Eames K, Bansal S, Frost S, Riley S. Six challenges in measuring contact networks for use in modeling. Epidemics. 2015;10:72–7. Epub 2015/04/07. doi: 10.1016/j.epidem.2014.08.006. PubMed PMID: 25843388.	Review or perspectives piece
Eames KTD, Tilston NL, Edmunds WJ. The impact of school holidays on the social mixing patterns of school children. Epidemics. 2011;3(2):103–8. doi: http://dx.doi.org/10.1016/j.epidem.2011.03.003. PubMed PMID: 361842166.	Data published elsewhere (Eames 2010)
Eames KTD. The influence of school holiday timing on epidemic impact. Epidemiology and Infection. 2014;142(9):1963–71. doi: http://dx.doi.org/10.1017/S0950268813002884. PubMed PMID: 373586411.	Modeling study
Edwards CH, Tomba GS, Blasio BFd. Influenza in workplaces: transmission, workers' adherence to sick leave advice and European sick leave recommendations. European Journal of Public Health. 2016;26(3):478–85. doi: http://dx.doi.org/10.1093/eurpub/ckw031. PubMed PMID: 20163190224.	Review or perspectives piece
Ewing A, Lee EC, Viboud C, Bansal S. Contact, travel, and transmission: The impact of winter holidays on influenza dynamics in the United States. Journal of Infectious Diseases. 2017;215(5):732–9. doi: http://dx.doi.org/10.1093/infdis/jiw642. PubMed PMID: 616354022.	Modeling study

Reference	Reason for Exclusion
Ferraro CF, Trotter CL, Nascimento MC, Jusot JF, Omotara BA, Hodgson A, et al. Household crowding, social mixing patterns and respiratory symptoms in seven countries of the African meningitis belt. PLoS ONE. 2014;9 (7) (no pagination)(e101129). doi: http://dx.doi.org/10.1371/journal.pone.0101129. PubMed PMID: 373459847.	Social contacts defined by attendance at events or involvement in activities
Fournet J, Barrat A. Contact patterns among high school students. PLoS ONE. 2014;9 (9) (no pagination)(e107878). doi: http://dx.doi.org/10.1371/journal.pone.0107878. PubMed PMID: 600033432.	Participants report contacts only with other study participants
Gerlier L, Weil-Olivier C, Carrat F, Lenne X, Lamotte M, Greneche S, et al. Public health and economic impact of vaccinating children with a quadrivalent live attenuated influenza vaccine in France using a dynamic transmission model. Value in Health. 2014;17 (7):A674. doi: http://dx.doi.org/10.1016/j.jval.2014.08.2502. PubMed PMID: 71674377.	Data published elsewhere (Mossong 2008)
Goeyvaerts N, Hens N, Ogunjimi B, Aerts M, Shkedy Z, Damme Pv, et al. Estimating infectious disease parameters from data on social contacts and serologic status. Journal of the Royal Statistical Society: Series C. 2010;59(2):255–77. doi: http://dx.doi.org/10.1111/j.1467-9876.2009.00693.x. PubMed PMID: 20103088230.	Data published elsewhere (Mossong 2008)
Guclu H, Read J, Vukotich CJ, Galloway DD, Gao H, Rainey JJ, et al. Social contact networks and mixing among students in K-12 Schools in Pittsburgh, PA. PLoS ONE. 2016;11 (3) (no pagination)(e0151139). doi: http://dx.doi.org/10.1371/journal.pone.0151139. PubMed PMID: 609076919.	Participants report contacts only within school
Hens N, Ayele GM, Goeyvaerts N, Aerts M, Mossong J, Edmunds JW, et al. Estimating the impact of school closure on social mixing behavior and the transmission of close contact infections in eight European countries. BMC Infectious Diseases. 2009;9 (no pagination)(187). doi: http://dx.doi.org/10.1186/1471-2334-9-187. PubMed PMID: 358047454.	Data published elsewhere (Mossong 2008)
Hens N, Goeyvaerts N, Aerts M, Shkedy Z, Van Damme P, Beutels P. Mining social mixing patterns for infectious disease models based on a two-day population survey in Belgium. BMC Infectious Diseases. 2009;9 (no pagination)(5). doi: http://dx.doi.org/10.1186/1471-2334-9-5. PubMed PMID: 354371756.	Data published elsewhere (Mossong 2008)
Huang C, Liu X, Sun S, Li SC, Deng M, He G, et al. Insights into the transmission of respiratory infectious diseases through empirical human contact networks. Sci Rep. 2016;6:31484. Epub 2016/08/17. doi: 10.1038/srep31484. PubMed PMID: 27526868; PubMed Central PMCID: PMCPMC4985757.	Participants report contacts only with other study participants
Kifle YW, Goeyvaerts N, Van Kerckhove K, Willem L, Faes C, Leirs H, et al. Animal ownership and touching enrich the context of social contacts relevant to the spread of human infectious diseases. PLoS ONE. 2015;10 (7) (no pagination)(e0133461). doi: http://dx.doi.org/10.1371/journal.pone.0133461. PubMed PMID: 606006430.	Data published elsewhere (Willem 2012)
Kiti MC, Tizzoni M, Kinyanjui TM, Koech DC, Munywoki PK, Meriac M, et al. Quantifying social contacts in a household setting of rural Kenya using wearable proximity sensors. EPJ data science. 2016;5:21. Epub 2016/07/30. doi: 10.1140/epjds/s13688-016-0084-2. PubMed PMID: 27471661; PubMed Central PMCID: PMCPMC4944592.	Participants report contacts only with other study participants
Kretzschmar M, Mikolajczyk RT. Contact profiles in eight European countries and implications for modeling the spread of airborne infectious diseases. PLoS ONE. 2009;4 (6) (no pagination)(e5931). doi: http://dx.doi.org/10.1371/journal.pone.0005931. PubMed PMID: 354877141.	Data published elsewhere (Mossong 2008)
Kretzschmar M, Teunis PFM, Pebody RG. Incidence and reproduction numbers of pertussis: Estimates from Serologic and Social Contact Data in Five European Countries. PLoS Medicine. 2010;7(6). doi: http://dx.doi.org/10.1371/journal.pmed.1000291. PubMed PMID: 359258160.	Data published elsewhere (Mossong 2008)
Kucharski AJ, Gog JR. The Role of Social Contacts and Original Antigenic Sin in Shaping the Age Pattern of Immunity to Seasonal Influenza. PLoS Computational Biology. 2012;8 (10) (no pagination)(e1002741). doi: http://dx.doi.org/10.1371/journal.pcbi.1002741. PubMed PMID: 365953585.	Data published elsewhere (Mossong 2008)
Kucharski AJ, Wenham C, Brownlee P, Racon L, Widmer N, Eames KTD, et al. Structure and consistency of self-reported social contact networks in British secondary schools. PLoS ONE. 2018;13(7):e0200090. doi: 10.1371/journal.pone.0200090.	Participants report contacts only within school
le Polain de Waroux O, Flasche S, Kucharski AJ, Langendorf C, Ndazima D, Mwanga-Amumpaire J, et al. Identifying human encounters that shape the transmission of *Streptococcus pneumoniae* and other acute respiratory infections. Epidemics. 2018.	Data published elsewhere (le Polain de Waroux 2018)
Leecaster M, Pettey W, Toth D, Rainey J, Uzicanin A, Samore M. Heterogeneity in social contact among school-age children and implications for influenza transmission. American Journal of Epidemiology. 2013;11):S151. doi: http://dx.doi.org/10.1093/aje/kwt103. PubMed PMID: 71079718.	Participants report contacts only with other study participants
Leecaster M, Toth DJA, Pettey WBP, Rainey JJ, Gao H, Uzicanin A, et al. Estimates of social contact in a middle school based on self-report and wireless sensor data. PLoS ONE. 2016;11 (4) (no pagination)(e0153690). doi: http://dx.doi.org/10.1371/journal.pone.0153690. PubMed PMID: 610063709.	Participants report contacts only with other study participants
Liccardo A, Fierro A. A Lattice Model for Influenza Spreading. PLoS ONE. 2013;8 (5) (no pagination)(e63935). doi: http://dx.doi.org/10.1371/journal.pone.0063935. PubMed PMID: 368973605.	Data published elsewhere (Mossong 2008)
Lowery-North DW, Hertzberg VS, Elon L, Cotsonis G, Hilton SA, Vaughns ICF, et al. Measuring Social Contacts in the Emergency Department. PLoS ONE. 2013;8 (8) (no pagination)(e70854). doi: http://dx.doi.org/10.1371/journal.pone.0070854. PubMed PMID: 369619793.	Participants report contacts only withemergency department patients and staff
Luca GD, Kerckhove KV, Coletti P, Poletto C, Bossuyt N, Hens N, et al. The impact of regular school closure on seasonal influenza epidemics: A data-driven spatial transmission model for Belgium. BMC Infectious Diseases. 2018;18 (1) (no pagination)(29). doi: http://dx.doi.org/10.1186/s12879-017-2934-3. PubMed PMID: 620158016.	Modeling study
Machens A, Gesualdo F, Rizzo C, Tozzi AE, Barrat A, Cattuto C. An infectious disease model on empirical networks of human contact: bridging the gap between dynamic network data and contact matrices. BMC	Participants report contacts only with other study participants

Reference	Reason for Exclusion
Infectious Diseases. 2013;13 (1) (no pagination)(185). doi: http://dx.doi.org/10.1186/1471-2334-13-185. PubMed PMID: 52561646.	
Melegaro A, Jit M, Gay N, Zagheni E, Edmunds WJ. What types of contacts are important for the spread of infections? Using contact survey data to explore European mixing patterns. Epidemics. 2011;3(3–4):143–51. doi: http://dx.doi.org/10.1016/j.epidem.2011.04.001. PubMed PMID: 51485516.	Data published elsewhere (Mossong 2008)
Meyer S, Held L. Incorporating social contact data in spatio-temporal models for infectious disease spread. Biostatistics (Oxford, England). 2017;18(2):338–51. doi: http://dx.doi.org/10.1093/biostatistics/kxw051. PubMed PMID: 617575085.	Data published elsewhere (Mossong 2008)
Milne GJ, Kelso JK, Kelly HA, Huband ST, McVernon J. A small community model for the transmission of infectious diseases: Comparison of School closure as an intervention in individual-based models of an influenza pandemic. PLoS ONE. 2008;3 (12) (no pagination)(e4005). doi: http://dx.doi.org/10.1371/journal.pone.0004005. PubMed PMID: 354011933.	Modeling study
Nguyen VK, Mikolajczyk R, Hernandez-Vargas EA. High-resolution epidemic simulation using within-host infection and contact data. BMC Public Health. 2018;18(1):886. doi: 10.1186/s12889-018-5709-x.	Modeling study
Ogunjimi B, Hens N, Goeyvaerts N, Aerts M, Damme Pv, Beutels P. Using empirical social contact data to model person to person infectious disease transmission: an illustration for varicella. Mathematical Biosciences. 2009;218(2):80–7. doi: http://dx.doi.org/10.1016/j.mbs.2008.12.009. PubMed PMID: 20093104437.	Data published elsewhere (Mossong 2008)
Oussaid N, Voirin N, Regis C, Khanafer N, Martin-Gaujard G, Vincent A, et al. Contacts between healthcare workers and patients in a short-stay geriatric unit during the peak of a seasonal influenza epidemic compared with a nonepidemic period. American Journal of Infection Control. 2016;44(8):905–9. doi: http://dx.doi.org/10.1016/j.ajic.2016.02.002. PubMed PMID: 609465419.	Participants report contacts only with other study participants
Ozella L, Gesualdo F, Tizzoni M, Rizzo C, Pandolfi E, Campagna I, et al. Close encounters between infants and household members measured through wearable proximity sensors. PLoS ONE. 2018;13 (6) (no pagination)(e0198733).	Participants report contacts only with other study participants
Potter GE, Handcock MS, Longini IM, Jr., Halloran ME. ESTIMATING WITHIN-HOUSEHOLD CONTACT NETWORKS FROM EGOCENTRIC DATA. The annals of applied statistics. 2011;5(3):1816–38. Epub 2011/01/01. PubMed PMID: 22427793; PubMed Central PMCID: PMCPMC3306235.	Participants report contacts only within school
Potter GE, Handcock MS, Longini IM, Jr., Halloran ME. ESTIMATING WITHIN-SCHOOL CONTACT NETWORKS TO UNDERSTAND INFLUENZA TRANSMISSION. The annals of applied statistics. 2012;6(1):1–26. Epub 2012/05/29. doi: 10.1214/11-aoas505. PubMed PMID: 22639701; PubMed Central PMCID: PMCPMC3359895.	Modeling study
Potter GE, Hens N. A penalized likelihood approach to estimate within-household contact networks from egocentric data. Journal of the Royal Statistical Society Series C, Applied statistics. 2013;62(4):629–48. Epub 2013/08/13. doi: 10.1111/rssc.12011. PubMed PMID: 23935218; PubMed Central PMCID: PMCPMC3736605.	Data published elsewhere (Mossong 2008)
Potter GE, Smieszek T, Sailer K. Modeling workplace contact networks: The effects of organizational structure, architecture, and reporting errors on epidemic predictions. Network science (Cambridge University Press). 2015;3(3):298–325. Epub 2015/12/04. doi: 10.1017/nws.2015.22. PubMed PMID: 26634122; PubMed Central PMCID: PMCPMC4663701.	Participants report contacts only with other study participants
Prem K, Cook AR, Jit M. Projecting social contact matrices in 152 countries using contact surveys and demographic data. PLoS Computational Biology. 2017;13 (9) (no pagination)(e1005697). doi: http://dx.doi.org/10.1371/journal.pcbi.1005697. PubMed PMID: 618570555.	Data published elsewhere (Mossong 2008)
Rainey JJ, Cheriyadat A, Radke RJ, Suzuki Crumly J, Koch DB. Estimating contact rates at a mass gathering by using video analysis: a proof-of-concept project. BMC public health. 2014;14:1101. doi: http://dx.doi.org/10.1186/1471-2458-14-1101. PubMed PMID: 605896131.	Methods paper
Read JM, Edmunds WJ, Riley S, Lessler J, Cummings DAT. Close encounters of the infectious kind: Methods to measure social mixing behavior. Epidemiology and Infection. 2012;140(12):2117–30. doi: http://dx.doi.org/10.1017/S0950268812000842. PubMed PMID: 366086476.	Review or perspectives piece
Salt P, Banner C, Oh S, Yu LM, Lewis S, Pan D, et al. Social mixing with other children during infancy enhances antibody response to a pneumococcal conjugate vaccine in early childhood. Clinical and Vaccine Immunology. 2007;14(5):593–9. doi: http://dx.doi.org/10.1128/CVI.00344-06. PubMed PMID: 352278830.	Social contacts defined by attendance at events or involvement in activities
Schmidt-Ott R, Schwehm M, Eichner M. Influence of social contact patterns and demographic factors on influenza simulation results. BMC Infectious Diseases. 2016;16 (1) (no pagination)(646). doi: http://dx.doi.org/10.1186/s12879-016-1981-5. PubMed PMID: 613266742.	Data published elsewhere (Mossong 2008)
Segerstrom SC. Social networks and immunosuppression during stress: Relationship conflict or energy conservation? Brain, Behavior, and Immunity. 2008;22(3):279–84. doi: http://dx.doi.org/10.1016/j.bbi.2007.10.011. PubMed PMID: 351172712.	Social contacts defined by attendance at events or involvement in activities
Smieszek T, Balmer M, Hattendorf J, Axhausen KW, Zinsstag J, Scholz RW. Reconstructing the 2003/2004 H3N2 influenza epidemic in Switzerland with a spatially explicit, individual-based model. BMC Infectious Diseases. 2011;11 (no pagination)(115). doi: http://dx.doi.org/10.1186/1471-2334-11-115. PubMed PMID: 51418223.	Modeling study
Smieszek T, Barclay VC, Seeni I, Rainey JJ, Gao H, Uzicanin A, et al. How should social mixing be measured: Comparing web-based survey and sensor-based methods. BMC Infectious Diseases. 2014;14 (1) (no pagination)(136). doi: http://dx.doi.org/10.1186/1471-2334-14-136. PubMed PMID: 372943011.	Participants report contacts only within school
Smieszek T, Burri EU, Scherzinger R, Scholz RW. Collecting close-contact social mixing data with contact diaries: reporting errors and biases. Epidemiology Infection. 2012;140(4):744–52.	Participants report contacts only with other study participants

Reference	Reason for Exclusion
Smieszek T, Castell S, Barrat A, Cattuto C, White PJ, Krause G. Contact diaries versus wearable proximity sensors in measuring contact patterns at a conference: Method comparison and participants' attitudes. BMC Infectious Diseases. 2016;16 (1) (no pagination)(341). doi: http://dx.doi.org/10.1186/s12879-016-1676-y. PubMed PMID: 611305281.	Participants report contacts only with other study participants
Stehle J, Voirin N, Barrat A, Cattuto C, Colizza V, Isella L, et al. Simulation of an SEIR infectious disease model on the dynamic contact network of conference attendees. BMC Medicine. 2011;9 (no pagination)(87). doi: http://dx.doi.org/10.1186/1741-7015-9-87. PubMed PMID: 51541345.	Participants report contacts only with other study participants
Stehle J, Voirin N, Barrat A, Cattuto C, Isella L, Pinton JF, et al. High-resolution measurements of face-to-face contact patterns in a primary school. PLoS ONE. 2011;6 (8) (no pagination)(e23176). doi: http://dx.doi.org/10.1371/journal.pone.0023176. PubMed PMID: 362343935.	Participants report contacts only with other study participants
Towers S, Feng Z. Social contact patterns and control strategies for influenza in the elderly. Mathematical Biosciences. 2012;240(2):241–9. doi: http://dx.doi.org/10.1016/j.mbs.2012.07.007. PubMed PMID: 52173631.	Data published elsewhere (Mossong 2008)
Vino T, Singh GR, Davison B, Campbell PT, Lydeamore MJ, Robinson A, et al. Indigenous Australian household structure: A simple data collection tool and implications for close contact transmission of communicable diseases. PeerJ. 2017;2017 (10) (no pagination)(e3958). doi: http://dx.doi.org/10.7717/peerj.3958. PubMed PMID: 618894679.	Participants report contacts only within household
Voirin N, Payet C, Barrat A, Cattuto C, Khanafer N, Regis C, et al. Combining high-resolution contact data with virological data to investigate influenza transmission in a tertiary care hospital. Infection Control and Hospital Epidemiology. 2015;36(3):254–60. doi: http://dx.doi.org/10.1017/ice.2014.53. PubMed PMID: 602525419.	Participants report contacts only with other study participants
Voirin N, Stehle J, Barrat A, Cattuto C, Isella L, Pinton JF, et al. Using wearable electronic sensors for assessing contacts between individuals in various environments. BMC Proceedings Conference: International Conference on Prevention and Infection Control, ICPIC. 2011;5(SUPPL. 6). PubMed PMID: 70730204.	Participants report contacts only with other study participants
Volz EM, Miller JC, Galvani A, Meyers L. Effects of heterogeneous and clustered contact patterns on infectious disease dynamics. PLoS Computational Biology. 2011;7 (6) (no pagination)(e1002042). doi: http://dx.doi.org/10.1371/journal.pcbi.1002042. PubMed PMID: 362058323.	Modeling study
Wallinga J, Edmunds WJ, Kretzschmar M. Perspective: Human contact patterns and the spread of airborne infectious diseases. Trends in Microbiology. 1999;7(9):372–7. doi: http://dx.doi.org/10.1016/S0966-842X%2899%2901546-2. PubMed PMID: 29421663.	Review or perspectives piece
Watson CH, Coriakula J, Ngoc DTT, Flasche S, Kucharski AJ, Lau CL, et al. Social mixing in Fiji: Who-eats-with-whom contact patterns and the implications of age and ethnic heterogeneity for disease dynamics in the Pacific Islands. PLoS ONE. 2017;12 (12) (no pagination)(e0186911). doi: http://dx.doi.org/10.1371/journal.pone.0186911. PubMed PMID: 619533637.	Participants report contacts only during meals
Willem L, Verelst F, Kuylen E, Abboud LA, Bicke J, Hens N, et al. Catching the risk for measles outbreaks in a clustered society. Tropical Medicine and International Health. 2017;22 (Supplement 1):52. doi: http://dx.doi.org/10.1111/%28ISSN%291365-3156. PubMed PMID: 618977811.	Data published elsewhere (Willem 2012)
Wood R, Racow K, Bekker LG, Morrow C, Middelkoop K, Mark D, et al. Indoor social networks in a south african township: Potential contribution of location to tuberculosis transmission. PLoS ONE. 2012;7 (6) (no pagination)(e39246). doi: http://dx.doi.org/10.1371/journal.pone.0039246. PubMed PMID: 365133365.	Data published elsewhere (Johnstone Robertson 2011)
Zagheni E, Billari FC, Manfredi P, Melegaro A, Mossong J, Edmunds WJ. Using time-use data to parameterize models for the spread of close-contact infectious diseases. American Journal of Epidemiology. 2008;168(9):1082–90. doi: http://dx.doi.org/10.1093/aje/kwn220. PubMed PMID: 352577381.	Social contacts defined by time use data

Appendix 1 Table 3. Publications Eligible for Inclusion That Did Not Collect (To Our Knowledge) Sex and Age Data for Participants and Contacts

Reference
Ajelli M, Litvinova M. Estimating contact patterns relevant to the spread of infectious diseases in Russia. Journal of Theoretical Biology. 2017 21 Apr;419:1–7.
Chan TC, Hu TH, Hwang JS. Estimating the risk for Influenza-Like Illness Transmission Through Social Contacts: Web-Based Participatory Cohort Study. JMIR public health and surveillance. 2018 Apr 9;4(2):e40.
Chen S-C, You S-H, Ling M-P, Chio C-P, Liao C-M. Use of seasonal influenza virus titer and respiratory symptom score to estimate effective human contact rates. Journal of epidemiology. 2012;22(4):353–63.
Danon L, House TA, Read JM, Keeling MJ. Social encounter networks: Collective properties and disease transmission. Journal of the Royal Society Interface. 2012 07 Nov;9(76):2826–33.
Destefano F, Haber M, Currivan D, Farris T, Burrus B, Stone-Wiggins B, et al. Factors associated with social contacts in four communities during the 2007–2008 influenza season. Epidemiology and Infection. 2011 August;139(8):1181–90.
Eames KTD, Tilston NL, Brooks-Pollock E, Edmunds WJ. Measured dynamic social contact patterns explain the spread of H1N1v influenza. PLoS Computational Biology. 2012 March;8 (3) (no pagination)(e1002425).
Edmunds WJ, O'Callaghan CJ, Nokes DJ. Who mixes with whom? A method to determine the contact patterns of adults that may lead to the spread of airborne infections. Proceedings of the Royal Society B: Biologic Sciences. 1997;264(1384):949–57.
Glass LM, Glass RJ. Social contact networks for the spread of pandemic influenza in children and teenagers. BMC Public Health. 2008;8 (no pagination)(61).
Ibuka Y, Ohkusa Y, Sugawara T, Chapman GB, Yamin D, Atkins KE, et al. Social contacts, vaccination decisions and influenza in Japan. Journal of epidemiology and community health. 2016 01 Feb;70(2):162–7.
Jackson C, Mangtani P, Vynnycky E, Fielding K, Kitching A, Mohamed H, et al. School closures and student contact patterns. Emerging infectious diseases. 2011;17(2):245.
Kiti MC, Kinyanjui TM, Koech DC, Munywoki PK, Medley GF, Nokes DJ. Quantifying age-related rates of social contact using diaries in a rural coastal population of Kenya. PLoS ONE. 2014 15 Aug;9 (8) (no pagination)(e104786).
Kucharski AJ, Kwok KO, Wei VWI, Cowling BJ, Read JM, Lessler J, et al. The Contribution of Social Behavior to the Transmission of Influenza A in a Human Population. PLoS Pathogens. 2014 June;10 (6) (no pagination)(e1004206).
Kwok KO, Cowling B, Wei V, Riley S, Read JM. Temporal variation of human encounters and the number of locations in which they occur: a longitudinal study of Hong Kong residents. Journal of the Royal Society, Interface. 2018 Jan;15(138).
Kwok KO, Cowling BJ, Wei VW, Wu KM, Read JM, Lessler J, et al. Social contacts and the locations in which they occur as risk factors for influenza infection. Proceedings. 2014 22 Aug;Biologic sciences / The Royal Society. 281(1789):20140709.
Lapidus N, De Lamballerie X, Salez N, Setbon M, Delabre RM, Ferrari P, et al. Factors associated with post-seasonal serologic titer and risk factors for infection with the pandemic A/H1N1 virus in the French general population. PloS one. 2013;8(4):e60127.
Read JM, Eames KTD, Edmunds WJ. Dynamic social networks and the implications for the spread of infectious disease. Journal of the Royal Society Interface. 2008 06 Sep;5(26):1001–7.
Read JM, Lessler J, Riley S, Wang S, Tan LJ, Kwok KO, et al. Social mixing patterns in rural and urban areas of southern China. Proceedings. 2014 22 Jun;Biologic sciences / The Royal Society. 281(1785):20140268.
Smieszek T. A mechanistic model of infection: why duration and intensity of contacts should be included in models of disease spread. Theoretical Biology and Medical Modeling. 2009;6(1):25.
Stein ML, van der Heijden PGM, Buskens V, van Steenbergen JE, Bengtsson L, Koppeschaar CE, et al. Tracking social contact networks with online respondent-driven detection: Who recruits whom? BMC Infectious Diseases. 2015;15 (1) (no pagination)(522).
Stein ML, Van Steenbergen JE, Buskens V, Van Der Heijden PGM, Chanyasanha C, Tipayamongkholgul M, et al. Comparison of contact patterns relevant for transmission of respiratory pathogens in Thailand and The Netherlands using respondent-driven sampling. PLoS ONE. 2014 25 Nov;9 (11) (no pagination)(e113711).
Stein ML, Van Steenbergen JE, Chanyasanha C, Tipayamongkholgul M, Buskens V, Van Der Heijden PGM, et al. Online respondent-driven sampling for studying contact patterns relevant for the spread of close-contact pathogens: A pilot study in Thailand. PLoS ONE. 2014 08 Jan;9 (1) (no pagination)(e85256).
Stromgren M, Holm E, Dahlstrom O, Ekberg J, Eriksson H, Spreco A, et al. Place-based social contact and mixing: A typology of generic meeting places of relevance for infectious disease transmission. Epidemiology and Infection. 2017 01 Sep;145(12):2582–93.
Wallinga J, Teunis P, Kretzschmar M. Using data on social contacts to estimate age-specific transmission parameters for respiratory-spread infectious agents. American Journal of Epidemiology. 2006 November;164(10):936–44.

Appendix 1 Table 4. Publications Eligible for Inclusion Known to Have Collected Sex and Age Data for Participants and Contacts

Reference
Beraud G, Kazmercziak S, Beutels P, Levy-Bruhl D, Lenne X, Mielcarek N, et al. The French connection: The first large population-based contact survey in France relevant for the spread of infectious diseases. PLoS ONE. 2015 15 Jul;10 (7) (no pagination)(e0133203).
Bernard H, Fischer R, Mikolajczyk RT, Kretzschmar M, Wildner M. Nurses' contacts and potential for infectious disease transmission. Emerging infectious diseases. 2009;15(9):1438.
Beutels P, Shkedy Z, Aerts M, Van Damme P. Social mixing patterns for transmission models of close contact infections: Exploring self-evaluation and diary-based data collection through a web-based interface. Epidemiology and Infection. 2006 December;134(6):1158–66.
Chen SC, You ZS. Social contact patterns of school-age children in Taiwan: Comparison of the term time and holiday periods. Epidemiology and Infection. 2015 15 Apr;143(6):1139–47.
Dodd PJ, Looker C, Plumb ID, Bond V, Schaap A, Shanaube K, et al. Age- and Sex-Specific Social Contact Patterns and Incidence of Mycobacterium tuberculosis Infection. American Journal of Epidemiology. 2016 15 Jan;183(2):156–66.
Eames KTD, Tilston NL, White PJ, Adams E, Edmunds WJ. The impact of illness and the impact of school closure on social contact patterns. Health Technology Assessment. 2010;14(34):267–312.
Edmunds W, Kafatos G, Wallinga J, Mossong J. Mixing patterns and the spread of close-contact infectious diseases. Emerging themes in epidemiology. 2006;3(1):10.
Fu Yc, Wang DW, Chuang JH. Representative Contact Diaries for Modeling the Spread of Infectious Diseases in Taiwan. PLoS ONE. 2012 03 Oct;7 (10) (no pagination)(e45113).
Grijalva CG, Goeyvaerts N, Verastegui H, Edwards KM, Gil AI, Lanata CF, et al. A household-based study of contact networks relevant for the spread of infectious diseases in the highlands of peru. PLoS ONE. 2015 03 Mar;10 (3) (no pagination)(e0118457).
Horby P, Thai PQ, Hens N, Yen NTT, Mai LQ, Thoang DD, et al. Social contact patterns in vietnam and implications for the control of infectious diseases. PLoS ONE. 2011;6 (2) (no pagination)(e16965).
Johnstone-Robertson SP, Mark D, Morrow C, Middelkoop K, Chiswell M, Aquino LDH, et al. Social mixing patterns within a South African township community: Implications for respiratory disease transmission and control. American Journal of Epidemiology. 2011 01 Dec;174(11):1246–55.
Kerckhove KV, Hens N, Edmunds WJ, Eames KTD. The impact of illness on social networks: Implications for transmission and control of influenza. American Journal of Epidemiology. 2013 01 Dec;178(11):1655–62.
Kumar S, Amarchand R, Gosain M, Sharma H, Dawood F, Jain S, et al. Design of a study to examine contact mixing and acute respiratory infection in Ballabgarh, Haryana. International Journal of Infectious Diseases. 2016 April;1):282.
le Polain de Waroux O, Cohuet S, Ndazima D, Kucharski AJ, Juan-Giner A, Flasche S, et al. Characteristics of human encounters and social mixing patterns relevant to infectious diseases spread by close contact: A survey in Southwest Uganda. BMC Infectious Diseases. 2018 11 Apr;18 (1) (no pagination)(172).
Leung K, Jit M, Lau EHY, Wu JT. Social contact patterns relevant to the spread of respiratory infectious diseases in Hong Kong. Sci Rep. 2017 Aug 11;7(1):7974.
Luh DL, You ZS, Chen SC. Comparison of the social contact patterns among school-age children in specific seasons, locations, and times. Epidemics. 2016 March 01;14:36–44.
McCaw JM, Forbes K, Nathan PM, Pattison PE, Robins GL, Nolan TM, et al. Comparison of three methods for ascertainment of contact information relevant to respiratory pathogen transmission in encounter networks. BMC infectious diseases. 2010;10(1):166.
Melegaro A, Fava ED, Poletti P, Merler S, Nyamukapa C, Williams J, et al. Social contact structures and time use patterns in the manicaland province of Zimbabwe. PLoS ONE. 2017 January;12 (1) (no pagination)(e0170459).
Mikolajczyk RT, Akmatov MK, Rastin S, Kretzschmar M. Social contacts of school children and the transmission of respiratory-spread pathogens. Epidemiology and Infection. 2008 June;136(6):813–22.
Mossong J, Hens N, Jit M, Beutels P, Auranen K, Mikolajczyk R, et al. Social contacts and mixing patterns relevant to the spread of infectious diseases. PLoS Medicine. 2008 March;5(3):0381–91.
Oguz MM, Camurdan AD, Aksakal FN, Akcaboy M, Altinel Acoglu E. Social contact patterns of infants in deciding vaccination strategy: A prospective, cross-sectional, single-center study. Epidemiology and Infection. 2018 01 Jul;146(9):1157–66.
Rolls DA, Geard NL, Warr DJ, Nathan PM, Robins GL, Pattison PE, et al. Social encounter profiles of greater Melbourne residents, by location–a telephone survey. BMC infectious diseases. 2015;15(1):494.
van de Kassteele J, van Eijkeren J, Wallinga J. Efficient estimation of age-specific social contact rates between men and women. The annals of applied statistics. 2017;11(1):320–39.
van Hoek AJ, Andrews N, Campbell H, Amirthalingam G, Edmunds WJ, Miller E. The Social Life of Infants in the Context of Infectious Disease Transmission; Social Contacts and Mixing Patterns of the Very Young. PLoS ONE. 2013 16 Oct;8 (10) (no pagination)(e76180).
Willem L, van Kerckhove K, Chao DL, Hens N, Beutels P. A Nice Day for an Infection? Weather Conditions and Social Contact Patterns Relevant to Influenza Transmission. PLoS ONE. 2012 14 Nov;7 (11) (no pagination)(e48695).

Appendix 1 Table 5. Contacts Reported by Boys and Girls with Boys, Girls, Men, and Women

Region	Survey	Participants	Contacts												
			Children						Adults						
			Boys		Girls		Total		Men		Women		Total		Total
			n	%	N	%	n	%	n	%	n	%	n	%	n
AFR	South Africa 2010	Boys	5.0	34	2.8	19	7.8	52	3.0	20	4.1	28	7.1	48	15.0
		Girls	3.1	19	6.2	39	9.2	58	2.4	15	4.3	27	6.7	42	15.9
	Zimbabwe 2013	Boys	1.6	17	2.4	26	4.0	43	2.0	22	3.3	36	5.3	57	9.3
		Girls	2.3	27	1.5	18	3.8	45	1.9	22	2.8	33	4.7	55	8.5
AMR	Peru 2011	Boys	6.2	32	4.0	21	10.2	53	4.2	22	4.9	25	9.1	47	19.3
		Girls	3.5	23	4.5	29	8.0	51	3.2	20	4.4	28	7.6	49	15.6
EUR	Belgium 2005–06	Boys	2.6	26	1.6	16	4.2	43	2.3	23	3.4	34	5.7	57	9.9
		Girls	1.9	16	2.8	24	4.7	40	2.9	25	4.1	35	7.0	60	11.7
	Belgium 2010–11	Boys	5.4	34	3.4	21	8.7	56	3.0	19	4.0	25	6.9	44	15.7
		Girls	3.6	20	6.1	34	9.7	55	2.9	17	5.1	29	8.0	45	17.7
	Finland 2005–06	Boys	4.5	35	2.6	20	7.2	56	2.4	19	3.3	26	5.7	45	12.9
		Girls	2.7	22	4.0	32	6.7	54	2.2	18	3.5	28	5.8	46	12.5
	France 2012	Boys	3.1	28	1.9	17	5.0	46	2.5	23	3.5	32	6.0	55	11.0
		Girls	2.3	19	3.2	26	5.5	45	2.6	21	4.2	34	6.8	55	12.3
	Germany 2005–06	Boys	2.0	24	1.1	13	3.1	38	2.1	26	3.0	37	5.1	62	8.2
		Girls	1.1	14	1.9	23	3.0	37	1.9	23	3.3	40	5.1	63	8.1
	Italy 2005–06	Boys	6.6	32	4.7	23	11.3	55	3.9	19	5.6	27	9.4	45	20.7
		Girls	5.0	24	7.0	34	12.0	58	3.4	16	5.4	26	8.8	42	20.7
	Luxembourg 2005–06	Boys	5.7	32	4.1	23	9.8	55	3.5	19	4.5	26	8.0	45	17.8
		Girls	4.2	26	4.9	30	9.1	56	3.0	18	4.3	26	7.3	45	16.4
	Netherlands 2005–06	Boys	6.2	39	3.9	25	10.1	64	2.7	17	3.1	19	5.8	36	15.9
		Girls	3.4	22	5.4	35	8.8	57	2.6	17	4.2	27	6.8	44	15.6
	Poland 2005–06	Boys	5.2	32	3.6	22	8.8	54	2.9	18	4.7	29	7.6	46	16.3
		Girls	3.3	20	4.7	29	8.0	49	3.3	20	5.1	31	8.4	51	16.3
	United Kingdom 2005–06	Boys	3.8	32	2.4	20	6.2	53	2.3	19	3.3	28	5.6	47	11.8
		Girls	2.6	19	4.7	35	7.2	54	2.2	16	4.0	30	6.2	46	13.5
EUR	United Kingdom 2012	Boys	0.7	12	0.5	9	1.2	21	1.7	29	2.9	50	4.6	79	5.8
		Girls	0.7	13	0.6	11	1.3	24	1.7	31	2.5	46	4.2	76	5.5
WPR	China 2010	Boys	6.3	40	3.3	21	9.6	60	2.7	17	3.6	23	6.3	40	15.8
		Girls	3.6	24	5.0	34	8.6	58	2.3	16	3.9	26	6.2	42	14.8
	China 2015–16	Boys	2.2	28	1.1	14	3.3	42	1.8	22	2.9	36	4.6	58	7.9
		Girls	0.8	12	1.5	24	2.3	36	1.4	22	2.6	42	4.0	64	6.3
	Vietnam 2007	Boys	2.2	33	1.2	18	3.5	51	1.6	23	1.8	26	3.3	49	6.8
		Girls	1.1	16	2.4	35	3.4	50	1.3	20	2.1	30	3.4	50	6.8

Appendix 1 Table 6. Sex-Assortative Mixing Reported by Boys and Girls in Contacts with Children and Adults

			Contacts			
			Children		Adults	
Region	Survey	Participants	%	95% CI	%	95% CI
AFR	South Africa 2010	Boys	64	(60–67)	42	(38–46)
		Girls	67	(64–70)	64	(60–68)
	Zimbabwe 2013	Boys	40	(38–42)	37	(36–39)
		Girls	40	(37–42)	60	(57–62)
AMR	Peru 2011	Boys	61	(58–63)	46	(44–49)
		Girls	56	(53–59)	57	(54–60)
EUR	Belgium 2005–06	Boys	62	(57–66)	41	(37–45)
		Girls	59	(55–63)	59	(55–62)
	Belgium 2010–11	Boys	62	(59–64)	43	(40–46)
		Girls	63	(60–65)	63	(61–66)
	Finland 2005–06	Boys	63	(60–66)	42	(39–46)
		Girls	60	(57–63)	61	(57–65)
	France 2012	Boys	62	(60–63)	42	(41–44)
		Girls	58	(56–60)	62	(60–63)
	Germany 2005–06	Boys	63	(59–68)	41	(37–45)
		Girls	65	(60–69)	63	(60–67)
	Italy 2005–06	Boys	59	(56–61)	41	(38–44)
		Girls	58	(55–61)	61	(58–64)
	Luxembourg 2005–06	Boys	58	(55–60)	43	(41–46)
		Girls	54	(51–57)	59	(56–62)
	Netherlands 2005–06	Boys	61	(59–64)	47	(43–51)
		Girls	61	(58–64)	62	(58–65)
	Poland 2005–06	Boys	59	(57–62)	38	(35–41)
		Girls	59	(56–62)	61	(58–63)
	United Kingdom 2005–06	Boys	62	(59–65)	41	(37–44)
		Girls	65	(62–67)	65	(62–68)
	United Kingdom 2012	Boys	55	(43–67)	37	(31–43)
		Girls	46	(34–59)	60	(53–66)
WPR	China 2010	Boys	66	(64–68)	43	(40–46)
		Girls	58	(56–61)	62	(59–66)
	China 2015–16	Boys	68	(63–73)	38	(34–42)
		Girls	66	(60–71)	64	(60–69)
	Vietnam 2007	Boys	64	(60–69)	47	(42–52)
		Girls	69	(64–73)	61	(56–65)

Appendix 1 Table 7. Contacts Reported by Boys and Girls with Boys, Girls, Men, and Women at Home and Outside the Home

			At Home										Outside the Home									
			Children				Adults						Children				Adults					
		Participants	Boys		Girls		Men		Women		Total		Boys		Girls		Men		Women		Total	
Region	Survey		n	%	n	%	n	%	n	%	n	%	n	%	n	%	n	%	n	%	n	%
AFR	South Africa 2010	Boys	0.9	6	0.7	5	1.3	9	2.2	15	5.1	34	4.1	27	2.1	14	1.7	11	2.0	13	9.9	66
		Girls	0.5	3	0.9	6	1.1	7	1.8	11	4.3	27	2.6	16	5.2	33	1.3	8	2.5	16	11.6	73
AMR	Peru 2011	Boys	1.6	8	1.4	7	1.9	10	2.4	13	7.3	38	4.6	24	2.6	14	2.3	12	2.4	13	11.9	62
		Girls	1.3	8	1.5	9	1.8	11	2.4	15	7.0	44	2.3	14	3.1	19	1.5	9	2.1	13	9.0	56
EUR	Belgium 2005–06	Boys	0.6	6	0.4	4	1.3	13	1.4	14	3.7	37	2.0	20	1.3	13	1.0	10	1.9	19	6.2	63
		Girls	0.6	5	0.4	3	1.3	11	1.4	12	3.7	32	1.3	11	2.3	20	1.6	14	2.7	23	7.9	68
	Finland 2005–06	Boys	0.8	6	0.7	5	1.2	9	1.2	9	3.9	30	3.7	29	1.9	15	1.2	9	2.1	16	8.9	70
		Girls	0.7	6	0.8	6	1.2	10	1.2	10	3.9	31	2.0	16	3.3	26	1.0	8	2.3	18	8.6	69
	France 2012	Boys	0.9	8	0.4	4	0.5	5	0.6	5	2.4	22	2.3	21	1.6	14	2.0	18	2.8	25	8.7	78
		Girls	0.5	4	0.6	5	0.4	3	0.7	6	2.2	18	1.9	15	2.6	21	2.2	18	3.5	28	10.2	82
	Germany 2005–06	Boys	0.4	5	0.3	4	1.2	15	1.7	21	3.6	44	1.6	20	0.8	10	0.9	11	1.3	16	4.6	56
		Girls	0.4	5	0.5	6	1.3	16	1.7	20	3.9	47	0.7	8	1.5	18	0.6	7	1.6	19	4.4	53
	Italy 2005–06	Boys	0.5	2	0.5	2	1.6	8	2.2	11	4.8	23	6.1	29	4.2	20	2.3	11	3.3	16	15.9	77
		Girls	0.4	2	0.5	2	1.5	7	1.9	9	4.3	21	4.6	22	6.5	31	1.9	9	3.6	17	16.6	79
	Luxembourg 2005–06	Boys	0.7	4	0.7	4	1.6	9	1.8	10	4.8	27	5.0	28	3.4	19	1.9	11	2.7	15	13.0	73
		Girls	0.6	4	0.5	3	1.4	9	1.5	9	4.0	24	3.6	22	4.4	27	1.6	10	2.8	17	12.4	76
	Netherlands 2005–06	Boys	0.8	5	0.6	4	1.3	8	1.3	8	4.0	25	5.3	33	3.3	21	1.5	9	1.8	11	11.9	75
		Girls	0.8	5	0.8	5	1.3	8	1.7	11	4.6	29	2.6	17	4.6	29	1.3	8	2.5	16	11.0	71
	Poland 2005–06	Boys	0.6	4	0.7	4	1.7	10	2.4	15	5.4	33	4.6	28	2.9	18	1.2	7	2.2	13	10.9	67
		Girls	0.6	4	0.7	4	1.8	11	2.5	15	5.6	34	2.7	16	4.0	24	1.5	9	2.6	16	10.8	66
	United Kingdom 2005–06	Boys	0.9	8	0.7	6	1.3	11	1.6	14	4.5	38	3.0	25	1.6	14	1.0	8	1.7	14	7.3	62
		Girls	0.8	6	1.1	8	1.2	9	1.8	13	4.9	36	1.8	13	3.6	27	1.0	7	2.2	16	8.6	64
	United Kingdom 2012	Boys	3.8	8	3.7	8	10.3	21	12.2	25	30.0	61	2.4	5	1.8	4	3.2	7	11.6	24	19.0	39
		Girls	4.1	9	3.2	7	8.5	18	10.1	22	25.9	55	2.8	6	2.9	6	5.3	11	10.0	21	21.0	45
WPR	China 2015–16	Boys	0.3	4	0.2	3	0.9	11	1.6	20	3.0	38	2.0	25	0.8	10	0.8	10	1.3	16	4.9	62
		Girls	0.3	5	0.2	3	0.9	14	1.4	22	2.8	44	0.5	8	1.3	20	0.6	9	1.2	19	3.6	56
WPR	Vietnam 2007	Boys	0.6	9	0.6	9	1.4	21	1.6	24	4.2	63	1.6	24	0.6	9	0.1	1	0.2	3	2.5	37
		Girls	0.6	9	0.6	9	1.3	19	1.7	25	4.2	62	0.5	7	1.7	25	0.1	1	0.3	4	2.6	38

Appendix 1 Table 8. Sex-Assortative Mixing Reported by Boys and Girls in Contacts with Children and Adults at Home and Outside the Home

			At Home				Outside the Home			
			Children		Adults		Children		Adults	
Region	Survey	Partici-pants	%	95% CI	%	95% CI	%	95% CI	%	95% CI
AFR	South Africa 2010	Boys	54	(45–62)	37	(32–43)	66	(62–70)	46	(41–52)
		Girls	67	(59–75)	62	(57–68)	67	(63–70)	65	(60–70)
AMR	Peru 2011	Boys	53	(48–58)	44	(40–48)	64	(61–67)	49	(45–53)
		Girls	53	(48–59)	57	(52–61)	57	(54–61)	58	(54–63)
EUR	Belgium 2005–06	Boys	62	(52–71)	48	(42–54)	61	(56–67)	35	(29–40)
		Girls	40	(31–50)	52	(46–58)	65	(60–69)	62	(58–67)
	Finland 2005–06	Boys	54	(47–60)	49	(44–54)	66	(63–69)	37	(33–42)
		Girls	52	(45–59)	49	(43–55)	62	(58–66)	70	(65–74)
	France 2012	Boys	69	(66–72)	45	(41–49)	59	(57–61)	41	(40–43)
		Girls	57	(54–61)	63	(59–66)	58	(56–60)	61	(60–63)
	Germany 2005–06	Boys	54	(43–64)	41	(36–46)	66	(61–71)	41	(36–47)
		Girls	54	(45–63)	57	(52–61)	69	(63–74)	73	(67–77)
	Italy 2005–06	Boys	52	(43–60)	41	(37–45)	59	(57–62)	41	(37–44)
		Girls	53	(43–63)	55	(50–60)	59	(56–61)	65	(61–69)
	Luxembourg 2005–06	Boys	49	(42–55)	47	(42–51)	59	(57–62)	41	(37–44)
		Girls	47	(39–55)	52	(47–57)	55	(52–58)	64	(60–68)
	Netherlands 2005–06	Boys	55	(48–63)	49	(43–54)	62	(59–66)	45	(40–51)
		Girls	50	(43–58)	56	(51–51)	63	(60–66)	66	(61–70)
	Poland 2005–06	Boys	45	(38–52)	40	(37–44)	62	(59–64)	36	(32–40)
		Girls	55	(48–63)	58	(54–52)	60	(57–63)	63	(59–67)
	United Kingdom 2005–06	Boys	55	(48–61)	44	(39–49)	64	(61–68)	38	(33–43)
		Girls	57	(51–63)	61	(57–66)	67	(64–70)	68	(64–72)
	United Kingdom 2012	Boys	51	(46–56)	46	(43–48)	56	(50–62)	22	(19–25)
		Girls	44	(39–49)	54	(51–58)	51	(45–57)	65	(62–68)
WPR	China 2015–16	Boys	52	(38–65)	37	(31–42)	71	(66–76)	40	(33–46)
		Girls	42	(29–56)	62	(56–68)	72	(64–77)	67	(61–74)
	Vietnam 2007	Boys	50	(42–58)	48	(42–53)	72	(66–77)	41	(26–57)
		Girls	52	(44–60)	58	(53–63)	78	(73–83)	80	(67–90)

Appendix 1 Table 9. Contacts Reported by Boys and Girls with Boys, Girls, Men, and Women at School and Elsewhere Outside the Home

			At School										Elsewhere Outside the Home									
			Children				Adults						Children				Adults					
		Participants	Boys		Girls		Men		Women		Total		Boys		Girls		Men		Women		Total	
Region	Survey		n	%	n	%	n	%	n	%	n	%	n	%	n	%	n	%	n	%	n	%
AFR	South Africa 2010	Boys	1.2	12	0.7	7	0.2	2	0.2	2	2.3	23	3.1	31	1.4	14	1.5	15	1.8	18	7.8	77
		Girls	1.3	11	2.2	18	0.2	2	0.5	4	4.2	35	1.4	12	3.2	26	1.2	10	2.1	17	7.9	65
AMR	Peru 2011	Boys	4.3	29	2.6	17	1.2	8	1.3	9	9.4	63	1.4	9	0.7	5	1.8	12	1.7	11	5.6	37
		Girls	2.5	21	3.5	29	1.0	8	1.2	10	8.2	68	0.6	5	0.6	5	1.1	9	1.6	13	3.9	32
EUR	Belgium 2005–06	Boys	1.2	17	0.8	11	0.1	1	0.4	6	2.5	36	1.1	16	0.6	9	1.0	14	1.8	26	4.5	64
		Girls	0.8	9	1.2	13	0.2	2	0.4	4	2.6	29	0.6	7	1.5	17	1.7	19	2.6	29	6.4	71
	Finland 2005–06	Boys	3.1	30	1.6	15	0.3	3	1.0	10	6.0	57	1.3	12	0.7	7	1.1	10	1.4	13	4.5	43
		Girls	1.7	16	2.6	25	0.3	3	1.0	9	5.6	53	0.7	7	1.4	13	1.0	9	1.9	18	5.0	47
	France 2012	Boys	0.1	1	0.0	0	0.0	0	0.0	0	0.1	1	2.2	26	1.5	17	2.0	23	2.8	33	8.5	99
		Girls	0.1	1	0.1	1	0.0	0	0.0	0	0.2	2	1.8	18	2.5	25	2.2	22	3.5	34	10.0	98
	Germany 2005–06	Boys	1.1	19	0.7	12	0.2	4	0.7	12	2.7	47	0.9	16	0.3	5	0.9	16	0.9	16	3.0	53
		Girls	0.6	11	1.1	20	0.1	2	0.8	15	2.6	47	0.3	5	0.8	15	0.6	11	1.2	22	2.9	53
	Italy 2005–06	Boys	4.4	26	3.7	22	0.7	4	1.7	10	10.5	62	2.1	12	0.8	5	1.7	10	1.9	11	6.5	38
		Girls	3.7	22	4.7	28	0.6	4	1.6	9	10.6	62	1.0	6	2.0	12	1.4	8	2.0	12	6.4	38
	Luxembourg 2005–06	Boys	3.7	26	2.7	19	0.7	5	1.2	8	8.3	58	1.9	13	1.1	8	1.3	9	1.8	13	6.1	42
		Girls	3.0	22	3.5	26	0.6	4	1.2	9	8.3	61	1.0	7	1.3	10	1.1	8	1.9	14	5.3	39
	Netherlands 2005–06	Boys	4.2	35	0.9	8	0.5	4	0.9	8	6.5	54	2.2	18	0.9	8	1.2	10	1.2	10	5.5	46
		Girls	2.0	16	3.3	27	0.5	4	1.0	8	6.8	56	0.9	7	1.8	15	1.0	8	1.7	14	5.4	44
	Poland 2005–06	Boys	4.5	33	2.8	21	0.2	1	1.0	7	8.5	63	1.2	9	0.8	6	1.3	10	1.7	13	5.0	37
		Girls	2.7	21	3.8	29	0.5	4	1.1	9	8.1	63	0.5	4	1.1	9	1.3	10	1.9	15	4.8	37
	United Kingdom 2005–06	Boys	2.8	32	1.4	16	0.3	3	0.8	9	5.3	60	0.8	9	0.5	6	0.9	10	1.3	15	3.5	40
		Girls	1.6	16	3.4	34	0.4	4	1.3	13	6.7	67	0.4	4	0.8	8	0.8	8	1.3	13	3.3	33
	United Kingdom 2012	Boys	0.0	0	0.0	0	0.1	3	0.2	7	0.3	10	0.3	10	0.2	7	0.5	17	1.7	57	2.7	90
		Girls	0.0	0	0.0	0	0.0	0	0.1	3	0.1	3	0.4	10	0.4	10	1.1	28	1.9	49	3.8	97
WPR	China 2015–16	Boys	1.9	28	0.8	12	0.3	4	0.6	9	3.6	53	0.8	12	0.4	6	0.9	13	1.1	16	3.2	47
		Girls	0.4	8	1.2	23	0.2	4	0.7	13	2.5	48	0.4	8	0.7	13	0.6	12	1.0	19	2.7	52
WPR	Vietnam 2007	Boys	3.3	60	1.2	22	0.2	4	0.2	4	4.9	89	0.1	2	0.1	2	0.1	2	0.3	5	0.6	11
		Girls	0.9	16	3.4	62	0.0	0	0.2	4	4.5	82	0.1	2	0.2	4	0.2	4	0.5	9	1.0	18

Appendix 1 Table 10. Sex-Assortative Mixing Reported by Boys and Girls in Contacts with Children and Adults at School and Elsewhere Outside the Home

			At School				Elsewhere Outside the Home			
			Children		Adults		Children		Adults	
Region	Survey	Participants	%	95% CI	%	95% CI	%	95% CI	%	95% CI
AFR	South Africa 2010	Boys	62	(54–69)	55	(38–71)	68	(64–73)	45	(40–51)
		Girls	62	(57–67)	69	(57–80)	70	(66–74)	65	(59–70)
AMR	Peru 2011	Boys	62	(59–66)	47	(41–53)	67	(61–73)	51	(45–56)
		Girls	58	(54–62)	55	(48–62)	52	(43–62)	61	(55–67)
EUR	Belgium 2005–06	Boys	60	(53–67)	25	(14–38)	63	(55–70)	36	(31–42)
		Girls	59	(52–66)	71	(57–82)	70	(63–76)	61	(56–66)
	Finland 2005–06	Boys	66	(62–70)	23	(17–31)	65	(59–71)	44	(39–50)
		Girls	60	(56–65)	80	(72–86)	67	(60–73)	65	(60–71)
	France 2012	Boys	70	(57–81)	42	(26–59)	59	(57–61)	41	(40–43)
		Girls	65	(56–74)	54	(39–69)	58	(56–60)	61	(60–63)
	Germany 2005–06	Boys	62	(55–69)	21	(14–31)	73	(65–80)	51	(44–58)
		Girls	66	(58–73)	87	(79–93)	73	(64–81)	66	(59–72)
	Italy 2005–06	Boys	55	(52–58)	31	(26–36)	73	(68–77)	47	(43–52)
		Girls	56	(53–59)	76	(68–80)	66	(61–71)	60	(54–65)
	Luxembourg 2005–06	Boys	57	(54–61)	37	(32–43)	63	(58–68)	43	(38–48)
		Girls	54	(51–57)	65	(59–71)	58	(52–63)	63	(58–68)
	Netherlands 2005–06	Boys	82	(78–85)	38	(30–47)	72	(67–77)	49	(43–46)
		Girls	62	(58–66)	68	(60–75)	66	(60–71)	65	(59–70)
	Poland 2005–06	Boys	62	(59–65)	15	(10–22)	61	(55–67)	44	(39–49)
		Girls	58	(55–62)	69	(62–75)	66	(59–73)	60	(55–65)
	United Kingdom 2005–06	Boys	66	(62–70)	28	(20–36)	60	(52–67)	43	(37–49)
		Girls	68	(64–71)	76	(70–82)	66	(58–73)	62	(56–68)
	United Kingdom 2012	Boys	50	(1–99)	18	(2–52)	62	(38–82)	22	(14–33)
		Girls	50	(1–99)	100	(16–100)	48	(27–69)	64	(53–75)
WPR	China 2015–16	Boys	72	(65–78)	28	(19–40)	68	(58–77)	44	(37–52)
		Girls	76	(67–83)	78	(67–87)	66	(55–75)	61	(52–69)
	Vietnam 2007	Boys	73	(67–78)	50	(28–72)	50	(23–77)	32	(14–55)
		Girls	79	(73–84)	91	(59–100)	68	(43–87)	78	(62–89)

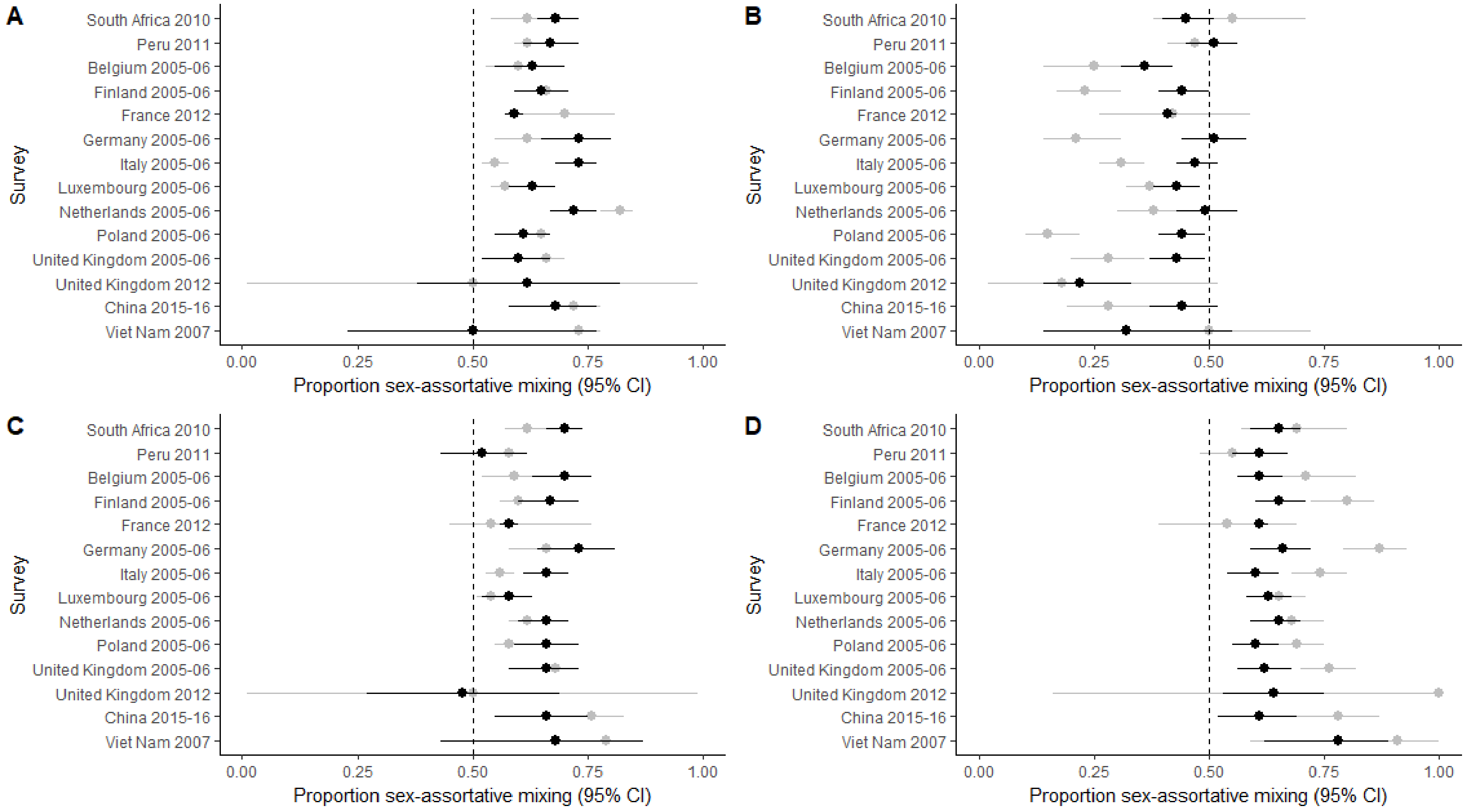

Appendix 1 Figure 1. Forest Plots of Sex-Assortative Mixing in Contacts Reported by Boys (A, B) and Girls (C, D) With Children (A, C) and With Adults (B, D) at School (Black) and Elsewhere Outside the Home (Grey). Plots show the proportion of contacts (with 95% confidence intervals) with the same sex, disaggregated by location, as reported for (A) boys with boys, (B) boys with men, (C) girls with girls, and (D) girls with women.

Appendix 1 Table 11. Contacts Reported by Men and Women with Boys, Girls, Men, and Women

			Contacts												
			Children						Adults						
			Boys		Girls		Total		Men		Women		Total		Total
Region	Survey	Participants	n	%	n	%	n	%	n	%	n	%	n	%	n
AFR	South Africa 2010	Men	0.8	6	0.7	5	1.5	10	7.9	52	5.7	38	13.6	90	15.1
		Women	1.2	7	1.6	9	2.7	16	5.5	33	8.4	51	13.9	84	16.7
	South Africa 2011	Men	0.4	7	0.3	7	0.7	14	2.5	50	1.8	36	4.3	86	5.0
		Women	0.6	10	0.7	13	1.3	23	1.6	28	2.7	49	4.3	77	5.5
	Zambia 2011	Men	0.2	5	0.3	5	0.5	10	2.9	59	1.5	31	4.4	90	4.9
		Women	0.4	8	0.4	8	0.7	16	1.3	27	2.7	57	4.0	84	4.7
	Zimbabwe 2013	Men	1.0	9	1.2	11	2.2	21	3.3	31	5.1	48	8.4	79	10.6
		Women	1.0	11	0.8	8	1.8	19	4.2	44	3.5	37	7.7	81	9.5
AMR	Peru 2011	Men	2.0	12	1.8	11	3.8	24	7.2	45	5.1	32	12.3	76	16.1
		Women	1.8	13	1.9	14	3.7	27	4.6	33	5.5	40	10.1	73	13.8
EUR	Belgium 2005–06	Men	0.3	3	0.3	3	0.6	6	6.2	53	5.0	42	11.2	95	11.8
		Women	0.6	5	0.7	6	1.3	11	4.7	39	6.1	51	10.8	89	12.0
	Belgium 2010–11	Men	0.4	3	0.4	3	0.9	7	6.6	51	5.5	42	12.1	93	13.0
		Women	0.6	5	0.6	5	1.2	10	4.8	38	6.6	52	11.4	90	12.6
	Finland 2005–06	Men	0.5	5	0.5	5	1.0	10	4.7	49	3.9	41	8.6	90	9.6
		Women	0.7	6	0.7	6	1.4	12	3.5	31	6.4	57	9.9	88	11.3
	France 2012	Men	0.3	3	0.2	2	0.5	5	5.3	51	4.6	44	9.9	95	10.4
		Women	0.4	4	0.4	4	0.8	8	4.3	41	5.4	51	9.7	92	10.5
	Germany 2005–06	Men	0.2	3	0.2	3	0.5	6	4.3	53	3.3	41	7.6	94	8.1
		Women	0.3	4	0.3	5	0.6	9	2.8	39	3.7	52	6.5	91	7.1
	Italy 2005–06	Men	0.9	4	0.5	2	1.3	7	10.3	53	7.9	40	18.2	93	19.5
		Women	1.3	7	1.3	7	2.5	14	6.8	37	9.0	49	15.8	86	18.3
	Luxembourg 2005–06	Men	0.6	4	0.4	3	1.0	6	9.5	55	6.7	39	16.2	94	17.2
		Women	1.3	8	1.3	8	2.6	15	6.5	38	8.1	47	14.6	85	17.1
	Netherlands 2005–06	Men	0.6	5	0.5	4	1.1	10	5.9	51	4.6	40	10.5	91	11.6
		Women	0.7	6	0.8	7	1.5	12	4.4	35	6.6	53	11.0	88	12.5
EUR	Poland 2005–06	Men	0.5	3	0.4	3	0.9	6	8.9	55	6.5	40	15.4	94	16.3
		Women	0.5	3	0.7	5	1.2	8	5.9	37	8.7	55	14.6	92	15.8
	United Kingdom 2005–06	Men	0.7	7	0.5	5	1.2	12	5.1	48	4.2	40	9.3	88	10.5
		Women	0.9	8	1.1	9	2.0	17	4.0	34	5.7	49	9.7	83	11.6
WPR	Australia 2008	Men	2.4	11	2.2	10	4.6	21	8.9	40	8.8	40	17.8	79	22.4
		Women	3.5	14	2.1	9	5.5	23	6.8	28	12.0	49	18.8	77	24.3
	Australia 2013	Men	0.3	5	0.2	4	0.5	9	2.3	43	2.6	48	4.9	91	5.4
		Women	0.3	6	0.4	7	0.7	12	2.1	36	3.0	52	5.1	88	5.8
	China 2010	Men	0.4	3	0.4	4	0.8	7	6.5	54	4.7	39	11.2	93	12.0
		Women	0.6	5	0.6	5	1.2	10	4.5	38	6.0	52	10.5	90	11.7
	China 2015–16	Men	0.3	4	0.2	3	0.4	7	2.7	45	2.9	48	5.6	93	6.0
		Women	0.3	5	0.3	5	0.6	10	2.2	33	3.8	57	6.0	91	6.6
	Vietnam 2007	Men	0.7	9	0.6	8	1.3	17	3.6	45	3.1	38	6.7	83	8.1
		Women	0.7	9	0.7	9	1.5	18	2.4	30	4.2	52	6.6	82	8.1

Appendix 1 Table 12. Sex-Assortative Mixing Reported by Men and Women in Contacts with Children and Adults

			Contacts			
			Children		Adults	
Region	Survey	Participants	%	95% CI	%	95% CI
AFR	South Africa 2010	Men	54	(48–60)	58	(56–60)
		Women	58	(53–62)	61	(59–62)
	South Africa 2011	Men	52	(47–56)	58	(56–60)
		Women	55	(51–58)	63	(61–65)
	Zambia 2011	Men	47	(43–51)	66	(64–67)
		Women	52	(49–55)	67	(65–68)
	Zimbabwe 2013	Men	45	(43–48)	39	(38–41)
		Women	47	(43–50)	45	(44–47)
AMR	Peru 2011	Men	53	(49–58)	59	(56–61)
		Women	51	(47–55)	54	(52–57)
EUR	Belgium 2005–06	Men	48	(40–56)	56	(54–58)
		Women	54	(49–60)	56	(55–58)
	Belgium 2010–11	Men	51	(47–55)	55	(54–56)
		Women	49	(46–53)	58	(57–59)
	Finland 2005–06	Men	53	(47–58)	55	(53–56)
		Women	50	(45–54)	64	(63–66)
	France 2012	Men	53	(48–58)	53	(52–54)
		Women	51	(48–54)	55	(55–56)
	Germany 2005–06	Men	51	(44–58)	57	(55–58)
		Women	53	(47–58)	57	(55–58)
	Italy 2005–06	Men	65	(60–70)	57	(55–58)
		Women	50	(46–53)	57	(56–58)
	Luxembourg 2005–06	Men	59	(53–64)	59	(57–60)
		Women	50	(47–53)	56	(54–57)
	Netherlands 2005–06	Men	57	(50–63)	56	(54–58)
		Women	54	(49–59)	60	(58–62)
	Poland 2005–06	Men	56	(50–61)	58	(56–59)
		Women	57	(52–61)	59	(58–61)
	United Kingdom 2005–06	Men	58	(53–63)	55	(53–57)
		Women	54	(51–58)	59	(57–60)
WPR	Australia 2008	Men	52	(44–60)	50	(46–54)
		Women	37	(34–41)	64	(62–66)
	Australia 2013	Men	54	(48–61)	47	(45–49)
		Women	52	(48–56)	58	(57–60)
	China 2010	Men	49	(45–53)	58	(57–59)
		Women	49	(45–52)	57	(56–58)
	China 2015–16	Men	59	(52–65)	48	(46–50)
		Women	48	(42–53)	64	(62–66)
	Vietnam 2007	Men	53	(47–58)	54	(52–56)
		Women	50	(46–55)	64	(62–66)

Appendix 1 Table 13. Contacts Reported by Men and Women with Boys, Girls, Men, and Women at Home and Outside the Home

			At Home										Outside the Home									
			Children				Adults						Children				Adults					
		Participants	Boys		Girls		Men		Women		Total		Boys		Girls		Men		Women		Total	
Region	Survey		n	%	n	%	n	%	n	%	n	%	n	%	n	%	n	%	n	%	n	%
AFR	South Africa 2010	Men	0.4	3	0.3	2	1.3	9	1.5	10	3.5	23	0.5	3	0.4	3	6.5	43	4.2	28	11.6	77
		Women	0.6	4	0.8	5	1.6	10	2.1	13	5.1	31	0.6	4	0.7	4	3.9	23	6.4	38	11.6	69
	South Africa 2011	Men	0.3	6	0.3	6	1.2	25	1.5	31	3.3	69	0.0	0	0.0	0	1.2	25	0.3	6	1.5	31
		Women	0.6	11	0.7	13	1.3	24	1.7	31	4.3	78	0.0	0	0.0	0	0.3	5	0.9	16	1.2	22
	Zambia 2011	Men	0.2	4	0.2	4	0.8	17	0.9	20	2.1	46	0.0	0	0.0	0	2.0	43	0.5	11	2.5	54
		Women	0.3	6	0.3	6	0.9	19	1.1	23	2.6	55	0.0	0	0.1	2	0.4	9	1.6	34	2.1	45
AMR	Peru 2011	Men	1.4	9	1.4	9	2.0	13	2.5	16	7.3	46	0.6	4	0.4	3	5.1	32	2.6	16	8.7	54
		Women	1.5	11	1.6	12	2.4	17	2.3	17	7.8	57	0.3	2	0.3	2	2.2	16	3.2	23	6.0	43
EUR	Belgium 2005–06	Men	0.2	2	0.3	3	1.0	8	1.6	13	3.1	26	0.1	1	0.1	1	5.2	44	3.4	29	8.8	74
		Women	0.3	2	0.4	3	1.5	12	1.1	9	3.3	27	0.3	2	0.3	2	3.2	26	5.0	41	8.8	73
	Finland 2005–06	Men	0.3	3	0.3	3	0.5	5	1.1	12	2.2	23	0.2	2	0.1	1	4.2	44	2.8	29	7.3	77
		Women	0.4	4	0.3	3	1.2	11	0.6	5	2.5	22	0.3	3	0.3	3	2.4	21	5.8	51	8.8	78
	France 2012	Men	0.1	1	0.0	0	1.3	12	0.7	7	2.1	20	0.2	2	0.2	2	4.0	38	4.0	38	8.4	80
		Women	0.1	1	0.1	1	0.7	7	0.7	7	1.6	15	0.3	3	0.4	4	3.7	35	4.7	44	9.1	85
	Germany 2005–06	Men	0.1	1	0.1	1	0.8	10	1.3	16	2.3	29	0.1	1	0.1	1	3.5	44	2.0	25	5.7	71
		Women	0.2	3	0.2	3	1.2	16	1.1	15	2.7	37	0.2	3	0.1	1	1.6	22	2.7	37	4.6	63
	Italy 2005–06	Men	0.3	2	0.2	1	0.9	5	1.7	9	3.1	16	0.5	3	0.3	2	9.3	48	6.2	32	16.3	84
		Women	0.4	2	0.4	2	1.6	9	1.5	8	3.9	21	0.9	5	0.9	5	5.2	28	7.6	41	14.6	79
	Luxembourg 2005–06	Men	0.3	2	0.2	1	1.1	6	1.7	10	3.3	19	0.3	2	0.2	1	8.3	49	5.0	29	13.8	81
		Women	0.4	2	0.3	2	1.7	10	1.3	8	3.7	21	0.9	5	1.0	6	4.8	28	6.9	40	13.6	79
	Netherlands 2005–06	Men	0.4	3	0.3	3	0.8	7	1.4	12	2.9	25	0.3	3	0.2	2	5.1	44	3.2	27	8.8	75
		Women	0.3	2	0.4	3	1.5	12	1.1	9	3.3	26	0.4	3	0.4	3	2.9	23	5.5	44	9.2	74
	Poland 2005–06	Men	0.3	2	0.3	2	1.4	9	2.1	13	4.1	25	0.2	1	0.1	1	7.4	46	4.4	27	12.1	75
		Women	0.3	2	0.4	3	1.8	11	1.9	12	4.4	28	0.3	2	0.3	2	4.1	26	6.8	43	11.5	72
	United Kingdom 2005–06	Men	0.4	4	0.4	4	0.9	8	1.5	14	3.2	30	0.3	3	0.1	1	4.3	41	2.7	25	7.4	70
		Women	0.5	4	0.5	4	1.6	14	1.3	11	3.9	34	0.4	3	0.5	4	2.4	21	4.3	37	7.6	66
WPR	China 2015–16	Men	0.2	3	0.1	2	0.5	8	1.1	18	1.9	31	0.1	2	0.1	2	2.2	36	1.8	30	4.2	69
		Women	0.2	3	0.2	3	0.8	12	0.8	12	2.0	30	0.2	3	0.1	1	1.4	21	3.0	45	4.7	70
	Vietnam 2007	Men	0.6	8	0.5	6	1.9	24	2.3	29	5.3	67	0.1	1	0.1	1	1.7	22	0.7	9	2.6	33
		Women	0.6	7	0.6	7	1.8	22	2.4	29	5.4	66	0.2	2	0.2	2	0.6	7	1.8	22	2.8	34

Appendix 1 Table 14. Sex-Assortative Mixing Reported by Men and Women in Contacts with Children and Adults at Home and Outside the Home

			At Home				Outside the Home			
			Children		Adults		Children		Adults	
Region	Survey	Participants	%	95% CI	%	95% CI	%	95% CI	%	95% CI
AFR	South Africa 2010	Men	54	(45–63)	47	(43–51)	62	(60–64)	61	(59–63)
		Women	59	(53–65)	57	(53–61)	56	(50–63)	62	(60–64)
	South Africa 2011	Men	52	(47–56)	45	(43–48)	75	(72–78)	80	(77–82)
		Women	54	(51–58)	58	(55–60)	74	(52–90)	75	(72–78)
	Zambia 2011	Men	45	(40–49)	47	(45–49)	79	(77–80)	79	(77–80)
		Women	51	(47–55)	55	(53–57)	55	(46–64)	79	(77–80)
AMR	Peru 2011	Men	51	(46–56)	45	(41–49)	59	(56–63)	67	(64–70)
		Women	52	(47–56)	49	(45–53)	49	(39–60)	59	(56–63)
EUR	Belgium 2005–06	Men	43	(34–53)	40	(36–44)	61	(59–63)	61	(58–63)
		Women	55	(48–62)	42	(39–46)	54	(46–61)	61	(59–63)
	Finland 2005–06	Men	50	(43–57)	32	(28–36)	71	(69–72)	60	(58–62)
		Women	49	(42–55)	36	(32–39)	51	(44–58)	71	(69–72)
	France 2012	Men	63	(52–74)	65	(63–67)	56	(55–57)	50	(49–52)
		Women	49	(42–55)	52	(49–54)	52	(48–55)	56	(55–57)
	Germany 2005–06	Men	45	(35–54)	39	(36–42)	62	(60–64)	63	(61–65)
		Women	57	(50–64)	46	(44–49)	48	(40–56)	62	(60–64)
	Italy 2005–06	Men	61	(52–69)	36	(32–40)	59	(58–61)	60	(58–61)
		Women	49	(43–55)	48	(45–51)	50	(46–54)	59	(58–61)
	Luxembourg 2005–06	Men	57	(50–65)	40	(37–43)	59	(58–60)	63	(61–64)
		Women	46	(40–52)	42	(40–45)	52	(48–55)	59	(58–60)
	Netherlands 2005–06	Men	54	(46–62)	36	(32–40)	66	(64–68)	62	(60–64)
		Women	56	(49–63)	41	(37–45)	52	(46–59)	66	(64–68)
	Poland 2005–06	Men	53	(46–60)	41	(38–44)	62	(61–64)	63	(61–64)
		Women	57	(51–64)	51	(48–53)	56	(49–63)	62	(61–64)
	United Kingdom 2005–06	Men	54	(48–60)	36	(33–40)	64	(62–66)	61	(59–63)
		Women	52	(47–57)	46	(43–49)	57	(51–62)	64	(62–66)
WPR	China 2015–16	Men	53	(44–62)	30	(27–34)	69	(66–71)	55	(53–58)
		Women	52	(44–60)	51	(47–54)	43	(35–51)	69	(66–71)
	Vietnam 2007	Men	53	(47–58)	45	(42–48)	74	(70–76)	69	(66–73)
		Women	49	(44–54)	58	(55–61)	54	(44–63)	74	(70–76)

Appendix 1 Table 15. Contacts Reported by Men and Women with Boys, Girls, Men, and Women at Work and Elsewhere Outside the Home

			At Work										Elsewhere Outside the Home									
			Children				Adults						Children				Adults					
			Boys		Girls		Men		Women		Total		Boys		Girls		Men		Women		Total	
Region	Survey	Participants	n	%	n	%	n	%	n	%	n	%	n	%	n	%	n	%	n	%	n	%
AFR	South Africa 2010	Men	0.0	0	0.1	1	1.4	12	0.9	7	2.4	20	0.5	4	0.3	2	5.4	45	3.5	29	9.7	80
		Women	0.1	1	0.1	1	0.5	4	0.7	6	1.4	11	0.5	4	0.7	6	3.6	30	6.0	49	10.8	89
	South Africa 2011	Men	0.0	0	0.0	0	0.5	22	0.1	4	0.6	26	0.0	0	0.0	0	1.3	57	0.4	17	1.7	74
		Women	0.0	0	0.0	0	0.1	5	0.4	19	0.5	24	0.0	0	0.0	0	0.4	19	1.2	57	1.6	76
	Zambia 2011	Men	0.0	0	0.0	0	0.4	12	0.1	3	0.5	15	0.0	0	0.0	0	2.2	67	0.6	18	2.8	85
		Women	0.0	0	0.0	0	0.1	3	0.9	23	1.0	25	0.1	3	0.1	3	0.6	15	2.2	55	3.0	75
AMR	Peru 2011	Men	0.0	0	0.0	0	0.9	9	0.3	3	1.2	12	0.7	7	0.5	5	5.2	50	2.8	27	9.2	88
		Women	0.0	0	0.0	0	0.1	1	0.2	3	0.3	4	0.4	5	0.4	5	2.7	35	3.9	51	7.4	96
EUR	Belgium 2005–06	Men	0.0	0	0.0	0	2.0	21	1.0	10	3.0	31	0.1	1	0.1	1	3.7	39	2.7	28	6.6	69
		Women	0.0	0	0.0	0	1.1	12	1.4	15	2.5	26	0.3	3	0.3	3	2.4	25	4.0	42	7.0	74
	Finland 2005–06	Men	0.0	0	0.0	0	2.0	24	1.0	12	3.0	37	0.2	2	0.1	1	2.7	33	2.2	27	5.2	63
		Women	0.1	1	0.1	1	0.9	9	2.4	25	3.5	36	0.3	3	0.3	3	1.7	18	3.9	40	6.2	64
	France 2012	Men	0.0	0	0.0	0	1.1	14	1.3	16	2.4	30	0.2	3	0.2	3	2.9	37	2.2	28	5.5	70
		Women	0.0	0	0.0	0	0.9	10	1.5	17	2.4	28	0.0	0	0.3	3	2.8	32	3.2	37	6.3	72
	Germany 2005–06	Men	0.0	0	0.0	0	1.8	27	0.8	12	2.6	39	0.1	1	0.1	1	2.3	34	1.6	24	4.1	61
		Women	0.0	0	0.0	0	0.6	11	1.1	20	1.7	31	0.2	4	0.1	2	1.3	24	2.1	39	3.7	69
	Italy 2005–06	Men	0.1	1	0.1	1	3.7	22	2.0	12	5.9	35	0.4	2	0.2	1	5.9	35	4.4	26	10.9	65
		Women	0.3	2	0.3	2	1.6	10	2.1	14	4.3	28	0.6	4	0.6	4	3.9	25	5.9	39	11.0	72
	Luxembourg 2005–06	Men	0.1	1	0.0	0	4.0	27	1.8	12	5.9	40	0.3	2	0.2	1	5.0	34	3.5	23	9.0	60
		Women	0.4	3	0.4	3	2.0	13	2.2	15	5.0	34	0.6	4	0.7	5	3.3	22	5.3	36	9.9	66
	Netherlands 2005–06	Men	0.0	0	0.0	0	2.5	26	1.0	10	3.5	36	0.3	3	0.2	2	3.2	33	2.6	27	6.3	64
		Women	0.1	1	0.1	1	1.0	10	1.8	18	3.0	29	0.4	4	0.3	3	2.2	22	4.3	42	7.2	71
	Poland 2005–06	Men	0.0	0	0.0	0	3.5	27	1.5	12	5.0	39	0.2	2	0.1	1	4.4	34	3.2	25	7.9	61
		Women	0.1	1	0.1	1	2.1	17	3.1	24	5.4	43	0.2	2	0.2	2	2.5	20	4.4	35	7.3	57
	United Kingdom 2005–06	Men	0.0	0	0.0	0	1.7	21	0.9	11	2.6	32	0.3	4	0.2	2	3.0	37	2.1	26	5.6	68
		Women	0.2	2	0.2	2	0.9	10	1.7	20	3.0	34	0.3	3	0.4	5	1.8	21	3.2	37	5.7	66
WPR	China 2015–16	Men	0.0	0	0.0	0	1.4	26	0.8	15	2.2	41	0.1	2	0.1	2	1.5	28	1.5	28	3.2	59
		Women	0.1	2	0.0	0	0.6	10	1.4	23	2.1	34	0.2	3	0.2	3	1.1	18	2.5	41	4.0	66
	Vietnam 2007	Men	0.0	0	0.0	0	1.6	34	0.5	11	2.1	45	0.2	4	0.1	2	1.4	30	0.9	19	2.6	55
		Women	0.0	0	0.0	0	0.4	8	1.2	25	1.6	33	0.3	6	0.3	6	0.7	15	1.9	40	3.2	67

Appendix 1 Table 16. Sex-Assortative Mixing Reported by Men and Women in Contacts with Children and Adults at Work and Elsewhere Outside the Home

			At Work				Elsewhere Outside the Home			
			Children		Adults		Children		Adults	
Region	Survey	Participants	%	95% CI	%	95% CI	%	95% CI	%	95% CI
AFR	South Africa 2010	Men	57	(48–65)	62	(57–67)	57	(48–65)	61	(58–63)
		Women	44	(26–62)	61	(54–67)	58	(51–65)	62	(60–64)
	South Africa 2011	Men	55	(32–77)	86	(81–90)	55	(62–77)	77	(74–80)
		Women	67	(9–99)	78	(71–84)	75	(51–91)	74	(71–78)
	Zambia 2011	Men	62	(48–75)	87	(83–90)	62	(48–75)	77	(75–79)
		Women	50	(1–99)	94	(92–96)	55	(46–64)	79	(78–81)
AMR	Peru 2011	Men	60	(51–68)	77	(69–84)	60	(51–68)	65	(62–68)
		Women	33	(1–91)	62	(47–76)	50	(40–60)	59	(56–63)
EUR	Belgium 2005–06	Men	63	(45–79)	67	(63–71)	63	(45–79)	57	(55–60)
		Women	59	(33–82)	56	(52–60)	53	(45–61)	63	(60–65)
	Finland 2005–06	Men	61	(50–71)	68	(64–71)	61	(50–71)	55	(52–58)
		Women	46	(30–63)	73	(71–76)	52	(45–59)	69	(67–71)
	France 2012	Men	50	(45–56)	46	(44–49)	50	(45–56)	52	(51–54)
		Women	51	(39–63)	64	(62–65)	92	(89–94)	53	(52–54)
	Germany 2005–06	Men	61	(50–72)	70	(67–72)	61	(50–72)	59	(57–62)
		Women	39	(17–64)	65	(61–68)	49	(40–57)	61	(59–63)
	Italy 2005–06	Men	69	(61–76)	65	(62–67)	69	(61–76)	57	(55–59)
		Women	49	(41–56)	57	(54–60)	51	(46–56)	60	(59–62)
	Luxembourg 2005–06	Men	60	(52–68)	69	(66–71)	60	(52–68)	59	(57–60)
		Women	50	(44–56)	52	(50–55)	53	(48–57)	62	(60–64)
	Netherlands 2005–06	Men	62	(52–71)	71	(68–74)	62	(52–71)	56	(53–59)
		Women	67	(51–80)	65	(62–69)	49	(41–56)	66	(64–68)
	Poland 2005–06	Men	64	(54–74)	70	(68–73)	64	(54–74)	58	(56–60)
		Women	52	(40–64)	60	(58–62)	58	(49–66)	64	(62–66)
	United Kingdom 2005–06	Men	66	(57–74)	66	(63–69)	66	(57–74)	59	(56–61)
		Women	55	(46–64)	65	(62–68)	58	(51–64)	64	(61–66)
WPR	China 2015–16	Men	68	(55–79)	63	(59–66)	68	(55–79)	50	(46–53)
		Women	18	(8–34)	68	(64–71)	51	(42–61)	69	(66–71)
	Vietnam 2007	Men	57	(41–72)	77	(72–82)	57	(41–72)	62	(57–68)
		Women	56	(21–86)	74	(69–79)	54	(44–63)	73	(69–77)

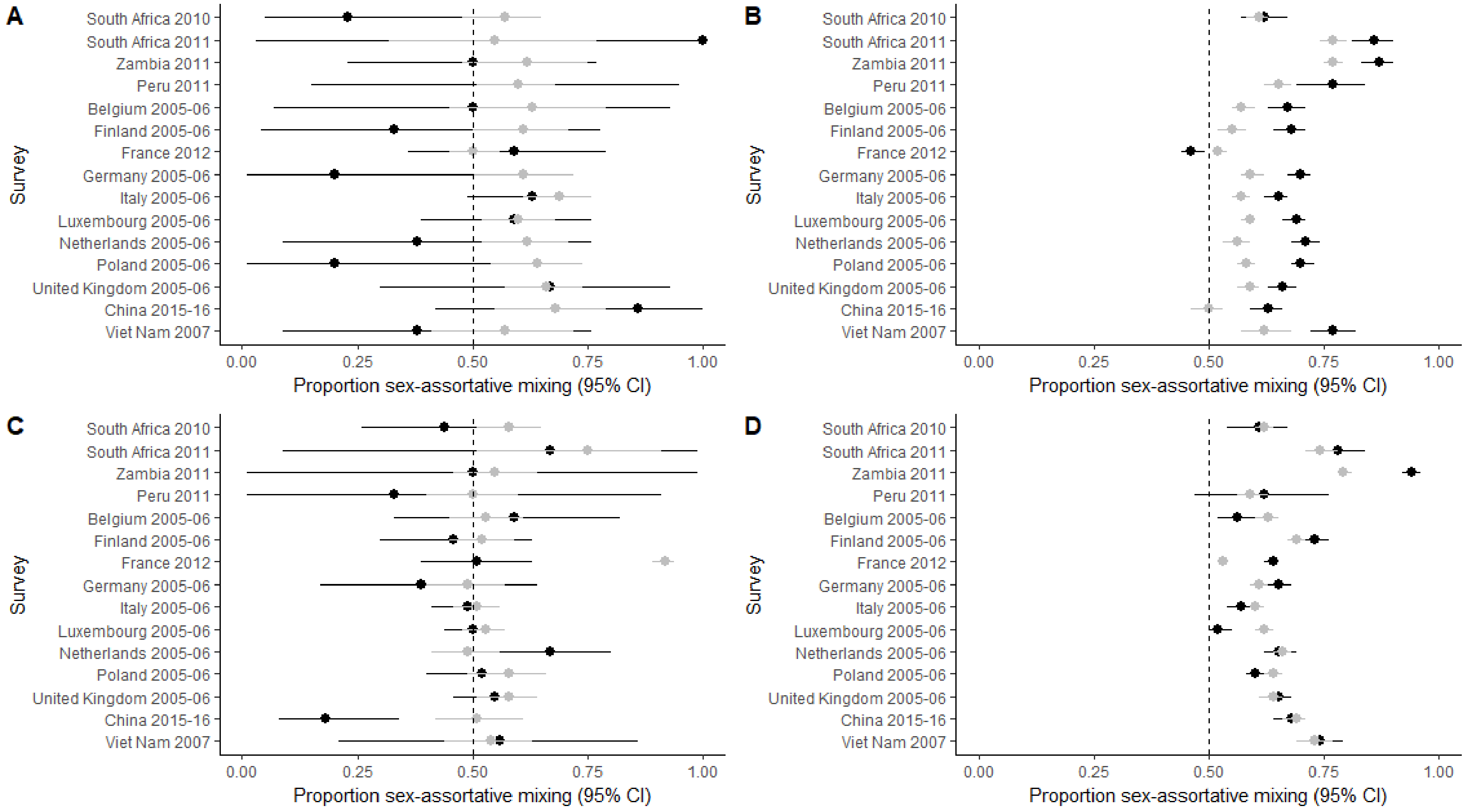

Appendix 1 Figure 2. Forest Plots of Sex-Assortative Mixing in Contacts Reported by Men (A, B) and Women (C, D) With Children (A, C) and With Adults (B, D) at Work (Black) and Elsewhere Outside the Home (Grey). Plots show the proportion of contacts (with 95% confidence intervals) with the same sex, disaggregated by location, as reported for (A) men with boys, (B) men with men, (C) women with girls, and (D) women with women.

Appendix 1 Table 17. Survey Characteristics Measured by the AXIS Tool

		Intro.				Methods				Results				Discussion				Oth.			
Region	Survey	Were the aims/ objectives of the study clear?	Was the study design appropriate for the stated aim(s) and aligned with understanding population-level social contact patterns?	Was the sample size justified?	Was the target/ reference population clearly defined and is that population the general population?	Was the sample frame taken from an appropriate population base so that it closely represented the target/ reference population under investigation?	Was the selection process likely to select subjects/ participants that were representative of the target/ reference population under investigation?	Were measures undertaken to address and categories non-responders?	Were the risk factor and outcome variables measured appropriate to the aims of the study?	Were the risk factor and outcome variables measured correctly using instruments that had been trialled, piloted or published previously?	Is it clear what was used to determined statistical significance and/or precision estimates?	Were the methods (including statistical methods) sufficiently described to enable them to be repeated?	Were the basic data adequately described?	Does the response rate raise concerns about non-response bias?	If appropriate, was information about non-responders described?	Were the results internally consistent?	Were the results for the analyses described in the methods, presented?	Were the authors' discussions and conclusions justified by the results?	Were the limitations of the study discussed?	Were there any funding sources or conflicts of interest that may affect the authors' interpretation of the results?	Was ethical approval or consent of participants attained?
AFR	South Africa 2010	Yes	Yes	No	Yes	Yes	Yes	No	Yes	Yes	Yes	Yes	Yes	No	No	Yes	Yes	Yes	Yes	No	Yes
	South Africa 2011	Yes	Yes	No	Yes	Yes	Yes	No	Yes	Yes	Yes	Yes	Yes	No	No	Yes	Yes	Yes	Yes	No	Yes
	Zambia 2011	Yes	Yes	No	Yes	Yes	Yes	No	Yes	Yes	Yes	Yes	Yes	No	No	Yes	Yes	Yes	Yes	No	Yes
	Zimbabwe 2013	Yes	Yes	No	Yes	Yes	Yes	No	Yes	Yes	Yes	Yes	Yes	Unk	No	Yes	Yes	Yes	Yes	No	Yes
AMR	Peru 2011	Yes	Yes	No	Yes	Yes	Yes	No	Yes	Yes	Yes	Yes	Yes	Unk	No	Yes	Yes	Yes	Yes	No	Yes
EUR	Belgium 2005–06	Yes	Yes	No	Yes	Yes	Yes	No	Yes	Yes	Yes	Yes	Yes	Unk	No	Yes	Yes	Yes	Yes	No	Yes
EUR	Belgium 2010–11	Yes	Yes	No	Yes	Yes	Yes	No	Yes	Yes	Yes	Yes	Yes	Unk	No	Yes	Yes	Yes	Yes	No	Yes
	Finland 2005–06	Yes	Yes	No	Yes	Yes	Yes	No	Yes	Yes	Yes	Yes	Yes	Unk	No	Yes	Yes	Yes	Yes	No	Yes
	France 2012	Yes	Yes	No	Yes	Yes	Yes	Yes	Yes	Yes	Yes	Yes	Yes	No	Yes	Yes	Yes	Yes	Yes	No	Yes
	Germany 2005–06	Yes	Yes	No	Yes	Yes	Yes	No	Yes	Yes	Yes	Yes	Yes	Unk	No	Yes	Yes	Yes	Yes	No	Yes
	Italy 2005–06	Yes	Yes	No	Yes	Yes	Yes	No	Yes	Yes	Yes	Yes	Yes	Unk	No	Yes	Yes	Yes	Yes	No	Yes
	Luxembourg 2005–06	Yes	Yes	No	Yes	Yes	Yes	No	Yes	Yes	Yes	Yes	Yes	Unk	No	Yes	Yes	Yes	Yes	No	Yes
	Netherlands 2005–06	Yes	Yes	No	Yes	Yes	Yes	No	Yes	Yes	Yes	Yes	Yes	Unk	No	Yes	Yes	Yes	Yes	No	Yes
EUR	Poland 2005–06	Yes	Yes	No	Yes	Yes	Yes	No	Yes	Yes	Yes	Yes	Yes	Unk	No	Yes	Yes	Yes	Yes	No	Yes

Region	Survey	Intro.				Methods				Results				Discussion				Oth.			
		Were the aims/ objectives of the study clear?	Was the study design appropriate for the stated aim(s) and aligned with understanding population-level social contact patterns?	Was the sample size justified?	Was the target/ reference population clearly defined and is that population the general population?	Was the sample frame taken from an appropriate population base so that it closely represented the target/ reference population under investigation?	Was the selection process likely to select subjects/ participants that were representative of the target/ reference population under investigation?	Were measures undertaken to address and categories non-responders?	Were the risk factor and outcome variables measured appropriate to the aims of the study?	Were the risk factor and outcome variables measured correctly using instruments that had been trialled, piloted or published previously?	Is it clear what was used to determined statistical significance and/or precision estimates?	Were the methods (including statistical methods) sufficiently described to enable them to be repeated?	Were the basic data adequately described?	Does the response rate raise concerns about non-response bias?	If appropriate, was information about non-responders described?	Were the results internally consistent?	Were the results for the analyses described in the methods, presented?	Were the authors' discussions and conclusions justified by the results?	Were the limitations of the study discussed?	Were there any funding sources or conflicts of interest that may affect the authors' interpretation of the results?	Was ethical approval or consent of participants attained?
	United Kingdom 2005–06	Yes	Yes	No	Yes	Yes	Yes	No	Yes	Yes	Yes	Yes	Yes	Unk	No	Yes	Yes	Yes	Yes	No	Yes
	United Kingdom 2012	Yes	Yes	No	Yes	Yes	Yes	No	Yes	Yes	Yes	Yes	Yes	Yes	Yes	Yes	Yes	Yes	Yes	No	Yes
WPR	Australia 2008	Yes	No	No	No	No	Unk	No	Yes	Yes	Yes	Yes	Yes	Unk	No	Yes	Yes	Yes	Yes	No	Yes
	Australia 2013	Yes	Yes	No	Yes	Yes	Yes	No	Yes	Yes	Yes	Yes	Yes	Yes	No	Yes	Yes	Yes	Yes	No	Yes
WPR	China 2010	Yes	Yes	Yes	Yes	Yes	Yes	No	Yes	Yes	Yes	Yes	Yes	No	No	Yes	Yes	Yes	Yes	No	Yes
	China 2015–16	Yes	Yes	No	Yes	Yes	Yes	No	Yes	Yes	Yes	Yes	Yes	Unk	No	Yes	Yes	Yes	Yes	No	Yes
	Vietnam 2007	Yes	Yes	No	Yes	Yes	Yes	No	Yes	Yes	Yes	Yes	Yes	Unk	No	Yes	Yes	Yes	Yes	No	Yes

Appendix 1 Table 18. Subgroup Analyses

	Proportion of adult contacts with men (random effects summary estimates)													
	Children							Adults						
		Boys			Girls				Men			Women		
Subgroup	n	%	95% CI	I^2	%	95% CI	I^2	n	%	95% CI	I^2	%	95% CI	I^2
Region														
African Region	2	39	(35–44)	78.9	38	(34–43)	79.0	4	55	(42–68)	99.6	41	(32–51)	99.2
Region of the Americas	1	46	(44–49)	-	43	(40–46)	-	1	59	(56–61)	-	46	(43–48)	-
European Region	11	42	(40–43)	47.5	38	(37–40)	26.4	10	56	(55–57)	84.1	42	(40–43)	92.6
Western Pacific Region	3	42	(38–47)	74.3	37	(35–40)	0.0	5	51	(46–57)	97.2	39	(36–42)	94.8
Setting														
National	10	42	(40–43)	51.1	39	(38–40)	24.0	9	56	(55–57)	84.9	42	(40–44)	93.4
Sub-national	7	42	(39–45)	85.5	38	(36–40)	64.2	11	54	(49–59)	98.9	40	(37–44)	98.0
Tuberculosis burden														
High	5	41	(38–44)	81.7	38	(36–40)	50.8	7	54	(47–62)	99.3	40	(35–45)	98.7
Low	12	42	(41–44)	62.1	39	(38–40)	46.5	13	55	92		42	(40–43)	93.2
Sampling														
Random	1	47	(42–52)	-	39	(35–44)	-	2	50	(43–58)	95.5	39	(34–44)	94.2
Stratified	4	41	(38–44)	82.7	38	(36–40)	61.8	6	56	(48–63)	99.4	41	(36–47)	98.7
Quota	11	41	(40–43)	53.1	39	(37–40)	27.4	10	55	(54–57)	91.7	41	(39–43)	94.4
Convenience	1	46	(44–49)	-	43	(40–46)	-	1	59	(56–61)	-	46	(43–48)	-
Unknown	0	-	(—)	-	-	(—)	-	1	50	(46–54)	-	36	(34–38)	-
Reporting duration														
24 h	15	42	(41–44)	62.0	38	(37–40)	46.0	17	56	(54–58)	95.6	40	(39–42)	94.6
48 h	2	40	(35–44)	93.6	39	(37–41)	59.3	2	46	(33–60)	99.6	50	(40–60)	99.1
72 h	0	-	(—)	-	-	(—)	-	1	50	(46–54)	-	36	(34–38)	-
Age of adult participants														
18+	0	-	(—)	-	-	(—)	-	3	57	(46–67)	99.1	37	(32–43)	96.8
16+	1	47	(42–52)	-	39	(35–44)	-	1	54	(52–56)	-	36	(34–38)	-
15+	14	42	(41–43)	56.7	38	(37–40)	48.0	15	56	(54–57)	90.6	41	(40–43)	93.7
13+	1	37	(36–39)	-	40	(38–42)	-	1	39	(38–41)	-	55	(53–56)	-
NA	1	37	(32–43)	-	40	(34–47)	-	0	-	(—)	-	-	(—)	-
Age of adult contacts														
16+	1	47	(42–52)	-	39	(35–44)	-	1	54	(52–56)	-	36	(34–38)	-
15+	15	42	(41–43)	57.6	38	(37–40)	44.7	16	55	(53–57)	93.4	41	(40–43)	93.3
13+	1	37	(36–39)	-	40	(38–42)	-	3	54	(37–70)	99.7	42	(29–55)	99.5
Participation														
Equitable	15	42	(40–43)	76.6	39	(38–40)	47.0	11	57	(54–59)	95.8	40	(37–42)	95.0
Excess males	2	42	(40–44)	0.1	38	(36–40)	0.0	1	39	(38–41)	-	55	(53–56)	-
Excess females	0	-	(—)	-	-	(—)	-	8	54	(52–56)	94.1	41	(40–43)	94.2
